# The origins and potential cross-species transmission of paramyxoviruses and other RNA viruses between native snakes and invasive Burmese pythons in the Florida Everglades

**DOI:** 10.1128/jvi.01833-25

**Published:** 2026-02-24

**Authors:** Ayusha Shrestha, Jasper W. Schwarz, Kurtis H. Feng, McKayla M. Spencer, Aastha Adhikari, Obed O. Amwe, Rita G. Sacharia, Bradley O'Hanlon, Thomas Denagamage, Alisia A. W. Weyna, Nicole M. Nemeth, Edward C. Holmes, Andrew B. Allison

**Affiliations:** 1 Department of Comparative, Diagnostic, and Population Medicine, College of Veterinary Medicine, University of Florida 684623, Gainesville, Florida, USA; 2 School of Medical Sciences, University of Sydney 216920, Sydney, New South Wales, Australia; 3 Florida Fish and Wildlife Conservation Commission 7033, Tallahassee, Florida, USA; 4 Department of Large Animal Clinical Sciences, College of Veterinary Medicine, University of Florida 374692, Gainesville, Florida, USA; 5 Southeastern Cooperative Wildlife Disease Study and Department of Pathology, College of Veterinary Medicine, University of Georgia 1355, Athens, Georgia, USA; Cornell University Baker Institute for Animal Health, Ithaca, New York, USA

**Keywords:** ferlavirus, alphavirus, hepacivirus, Burmese python, Everglades, invasive species

## Abstract

During November–December 2020, a disease outbreak involving eastern mudsnakes (*Farancia abacura abacura*) was documented in the Greater Everglades Ecosystem, Florida, USA. Moribund snakes were observed writhing on riverbanks with concomitant frothy oral discharge. Pathological examination revealed necrotizing enteritis and pulmonary edema, and a reptilian ferlavirus phylogenetically related to anaconda paramyxoviruses was isolated. While ferlaviruses are well-described pathogens of snakes in zoological and private collections worldwide, this represented the first outbreak of ferlaviruses recorded in free-ranging snakes. To biologically characterize this virus, we performed *in vitro* analyses including host range, relative infectivity, and temperature-dependent fitness assays. As one outstanding question in paramyxovirus biology is the functional role of the U protein, which is unique to ferlaviruses, we investigated its spatiotemporal expression in infected cells, demonstrating its localization to viral inclusion bodies. Additionally, we sampled Burmese pythons (*Python molurus bivittatus*), an invasive constrictor species, in and around the outbreak site in the Everglades for reptilian ferlaviruses, resulting in the discovery of the first terrestrial alphavirus detected in the United States in over 50 years and a novel hepacivirus that putatively contains a third protease which is evolutionarily conserved among lower vertebrate hepaciviruses and may have arisen by duplication of the NS2-3 protease. Although the impact of Burmese pythons on the Everglades ecosystem via the predation of native species has garnered much attention, little is known regarding the diversity of viral pathogens they harbor and the potential risks they may pose to native wildlife and other animal hosts through viral cross-species transmission.

**IMPORTANCE:**

Although the evolution, molecular biology, and pathogenesis of numerous disease-causing animal paramyxoviruses have been examined extensively, studies on reptilian ferlaviruses—which have been responsible for large-scale mortality events in snakes in managed settings for decades—have lagged significantly. Herein, we document the first recognized ferlavirus disease outbreak in free-ranging snakes and provide new insights on the ferlavirus-specific U protein and reptilian host range that make these viruses unique among the paramyxoviruses. In searching for the potential source of ferlaviruses in nature, we discovered other novel RNA viruses in Burmese pythons, an invasive constrictor species introduced into the Everglades. In addition to extending the known diversity of reptilian viruses, these findings provide new fundamental insights into mammalian virus evolution and highlight the potential dangers of the cross-species transmission of such viruses into native snakes and other wildlife.

## INTRODUCTION

Paramyxoviruses are a group of enveloped, non-segmented, negative-sense RNA viruses that contain numerous viruses of public health significance, such as measles and Nipah viruses, but also important veterinary pathogens, including canine distemper and Newcastle disease viruses. Currently, the family *Paramyxoviridae* is divided into four subfamilies—*Avulavirinae*, *Rubulavirinae*, *Metaparamyxovirinae*, and *Orthoparamyxovirinae* (1). The orthoparamyxoviruses are the most diverse subfamily, containing eight genera that infect a wide range of hosts, including mammals (*Henipavirus*, *Jeilongvirus*, *Morbillivirus*, *Respirovirus*, *Narmovirus, Salemvirus*), fish (*Aquaparamyxovirus*), and reptiles (*Ferlavirus*). The genus *Ferlavirus* currently contains a single classified species, *Ferlavirus reptilis*, commonly known as fer-de-lance virus (FDLV) or reptilian ferlavirus. FDLV was originally isolated from captive fer-de-lances (*Bothrops atrox*)—a pit viper species native to South America—in a serpentarium in Switzerland in 1972 (2). Since that time, ferlavirus infections and associated outbreaks of severe and often fatal disease have been described worldwide in zoological and private collections of snakes, making ferlaviruses an important pathogen to the captive reptile industry, snake farms, and conservation or anti-venom programs (3, 4).

Ferlavirus infection in snakes predominantly targets the respiratory tract (e.g., interstitial pneumonia, pulmonary edema) (5–7), and to a lesser extent, the central nervous system (e.g., meningoencephalitis, axonal demyelination) (5, 7). Clinical signs are varied and may include respiratory distress, oral discharge, regurgitation, abnormal posturing, convulsions, and anorexia (3, 6, 8). Infection may culminate in systemic disease and death, although disease development and outcomes are likely multifaceted and may depend upon the age and species of snake, ferlavirus strain, underlying conditions or co-infections, and immunocompetency among other factors (3, 5, 6, 9–11). Historically, outbreaks have been most often associated with members of the family *Viperidae* (e.g., true vipers, rattlesnakes), but many different families are now known to be susceptible to disease, including *Boidae* (e.g., boas), *Pythonidae* (e.g., pythons), *Colubridae* (e.g., corn snakes), and *Elapidae* (e.g., cobras) (6, 10, 12–14). In addition, periodic infections have been reported in a number of lizard and chelonian (tortoise) species, demonstrating a potentially wide host range across the class *Reptilia* (15–19). In contrast to the numerous reported outbreaks of ferlaviruses in snakes in managed settings, knowledge on ferlavirus distribution among free-ranging snakes remains exceedingly limited (3, 20, 21).

Depending upon the genus, paramyxovirus genomes contain 6–8 genes, which are flanked by 3′ leader and 5′ trailer sequences (1). Most paramyxoviruses contain six genes, which encode the nucleocapsid (N) protein, phosphoprotein (P), matrix (M) protein, fusion (F) protein, receptor-binding protein (RBP; further delineated into hemagglutinin [H], hemagglutinin-neuraminidase [HN], or glycoprotein [G]), and large (L) protein or RNA-dependent RNA polymerase (3′-N-P-M-F-RBP-L-5′). The P ORF may also produce additional proteins, such as the V protein through mRNA editing, C proteins through leaky ribosomal scanning, or Y proteins through ribosomal shunting (22). Ferlaviruses deviate from the canonical six-gene format, as they have an additional ORF between N and P which encodes the U protein, with the “U” designation indicating that its function is unknown (23). In addition, ferlaviruses (and Salem virus) are unique among the orthoparamyxoviruses in that the P ORF normally encodes for the non-essential V protein and is thus more similar to rubulaviruses in that respect (24).

Here, we report on the pathological findings of a reptilian ferlavirus outbreak in eastern mudsnakes (*Farancia abacura abacura*) that occurred in the Everglades and Francis S. Taylor Wildlife Management Area, Florida, USA. As this was the first recorded ferlavirus outbreak and mortality event in wild snakes, we characterized the mudsnake virus isolate through *in vitro* analyses in reptilian and mammalian cells to evaluate host range, relative infectivity, temperature-dependent fitness, and mRNA editing of the P gene. As U protein expression has not been visualized in infected cells, we examined its spatial localization over time, demonstrating its association with N and P in inclusion bodies. In search of the source of the outbreak virus in nature, we also investigated whether the ferlavirus could be found in introduced, nonnative invasive snakes (i.e., Burmese pythons [*Python molurus bivittatus*]) in and around the mudsnake ferlavirus outbreak site. This resulted in the discovery of a number of RNA viruses of potential ecological and evolutionary significance.

## RESULTS

### Ferlavirus outbreak in mudsnakes

**Fig 1:**
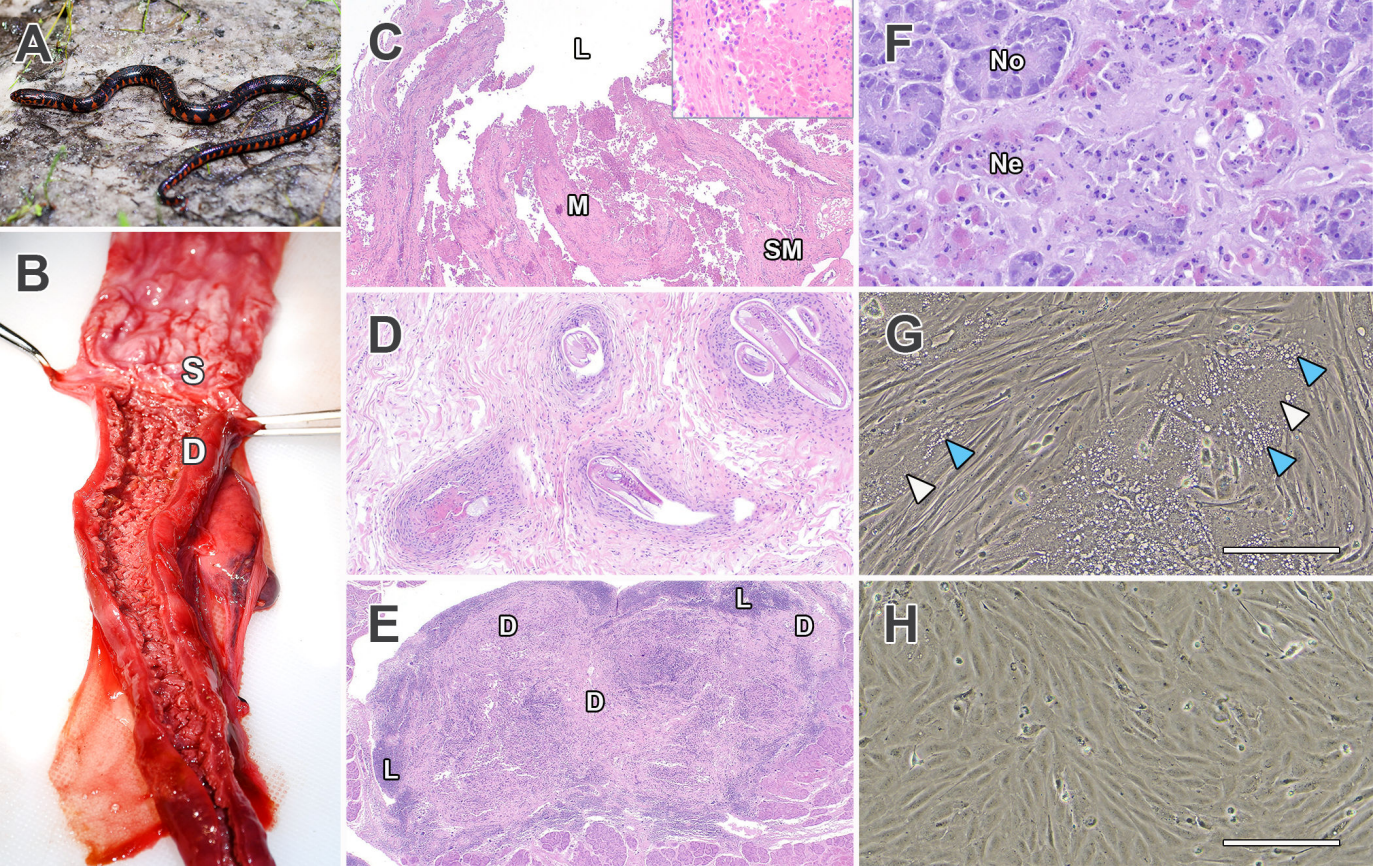
Gross and histological lesions in free-ranging eastern mudsnakes infected with reptilian ferlavirus. (**A**) Juvenile eastern mudsnake (*Farancia abacura abacura*). Photo courtesy of Steven Klioze, published with permission. (**B**) Intestine: severe, diffuse thickening and transmural reddening of the intestinal mucosa in mudsnake A. The adjacent stomach mucosa is also inflamed (S = stomach; D = duodenum). (**C**) Intestine: severe, diffuse, necroheterophilic enteritis in mudsnake A (L = lumen; M = mucosa; SM = submucosa) (hematoxylin and eosin [H&E] stain, 2× magnification). Inset: abundant sloughed and necrotic epithelial cells and pyknotic debris overlying the mucosal epithelium (H&E, 20× magnification). (**D**) Stomach: cross-sections of nematodes in the gastric muscular layer in mudsnake A, with surrounding lymphocytes and epithelioid macrophages (H&E stain, 4× magnification). (**E**) Spleen: severe lymphoid depletion in mudsnake A (D = lymphoid depleted areas; L = remaining lymphoid tissue) (H&E stain, 2× magnification). (**F**) Pancreas: multifocal necrosis of acinar epithelial cells in mudsnake B (Ne = necrotic epithelial cells; No = normal epithelial cells) (H&E stain, 20× magnification). (**G**) Russell’s viper heart (VH2) cells infected with the reptilian ferlavirus isolated from the intestinal tract of mudsnake A (isolate 21-Fa12-A). Note the characteristic multi-nucleated cells or syncytia (white arrowheads), which are associated with congregations of small membranous vesicles/vacuoles, presumably derived from the fusion process (blue arrowheads). Scale bar = 250 µm. (**H**) Control non-infected VH2 cells. Scale bar = 250 µm.

Approximately 15–20 eastern mudsnakes—a native, non-venomous, aquatic snake species (25) (Fig. 1A)—were observed sick or dead by Florida Fish and Wildlife Conservation Commission (FWC) python removal contractors on multiple occasions during November and December 2020 along the banks of Levees 28 and 67-A (L-28 and L67-A) within the Everglades and Francis S. Taylor Wildlife Management Area, Florida, USA. Snakes observed alive were often seen writhing and contorting on the riverbanks, with several of the snakes having a frothy liquid oral discharge. Two snakes that were found dead in December 2020 along L67-A were submitted to the Southeastern Cooperative Wildlife Disease Study (SCWDS) for diagnostic evaluation and were designated with case identifiers W21-012-A (Snake A) and W21-012-B (Snake B).

### Gross and histopathological examination of mudsnakes from the outbreak

Gross lesions in snake A were most prominent in the gastrointestinal tract, lungs, and skin. The gastrointestinal wall was severely thickened and transmurally reddened (Fig. 1B). The lungs were also diffusely erythematous with thickened airways. The skin over the dorsal head and neck had few, mild foci of wrinkled or displaced scales or crusts. Gross lesions in snake B were more limited and included multiple, widely scattered foci of dull, flaky scales along both sides of the body.

Histopathologic interpretation in both snakes was complicated by freeze-thaw and autolytic artifacts due to decomposition, and numerous tissues had widely scattered bacterial bacilli (interpreted as postmortem bacterial proliferation). Histologic lesions in both snakes A and B were most prominent in the intestinal tract and consisted of moderate, subacute multifocal, heterophilic, and necrotizing enteritis with extensive mucosal blunting and mild hemorrhage (Fig. 1C). Both snakes had diffuse, marked pulmonary edema and vascular congestion (with rare mild hemorrhage), along with pulmonary epithelial hyperplasia (with rare mild pneumocyte necrosis). Gastrointestinal tract lesions specific to snake A included adult nematodes in the gastric and intestinal muscular layer (Fig. 1D). Nematodes were within cyst-like spaces and surrounded by small numbers of epithelioid macrophages and lymphocytes, and a moderately thick band of fibrous connective tissue. The splenic portion of the splenopancreas in snake A was markedly depleted of lymphoid tissue with randomly scattered pyknotic foci (Fig. 1E). Lesions specific to snake B included a few contiguous clusters of acini with necrotic epithelial cells in the pancreatic portion of the splenopancreas (Fig. 1F). Skin over the face and body had multifocal, irregular hyperkeratosis with overlying serocellular crusts.

Ancillary RT-PCR or PCR testing for serpentoviruses and *Ophidiomyces ophidiicola*, the etiologic agent of snake fungal disease (ophidiomycosis), was negative in both snakes. Aerobic bacterial culture of liver samples resulted in light growth of *Aeromonas hydrophila* (snake A) or *Providencia rettgeri* and *Citrobacter braakii* (snake B)*,* which were interpreted as not clinically relevant based on minimal growth, lack of a predominant pathogenic organism, and absence of compatible histologic lesions in the liver.

### Virus isolation and genomic analysis

A reptilian ferlavirus was isolated from the intestinal tract and kidney of snake A (hereafter referred to as isolate 21-Fa12-A) (Fig. 1G). No virus was isolated from snake B. As the two snakes were found dead (with unknown postmortem intervals), the lack of consistent isolation from tissues was not surprising. RNA-Seq analysis of isolate 21-Fa12-A from intestinal tissue demonstrated that the genome was 15,378 nt in length and most similar (97.7%–97.8% nucleotide identity) to paramyxoviruses isolated from green anacondas (*Eunectes murinus*) in a Hong Kong oceanarium from 2011 to 2012 and 2015 (9, 26), in addition to ferlaviruses recently identified in caiman lizards (*Dracaena guianensis*) imported into France and Germany from Peru (97.7%) (19). Similarly close nucleotide identities were observed in short U or L gene sequences (<600 nt) to other snake and tortoise viruses, which lack genomic data (e.g., GenBank accession GQ277627, 97.4% in U; GU726898, 97.3% in L) (17, 27).

### Evolutionary analysis of the mudsnake ferlavirus

**Fig 2:**
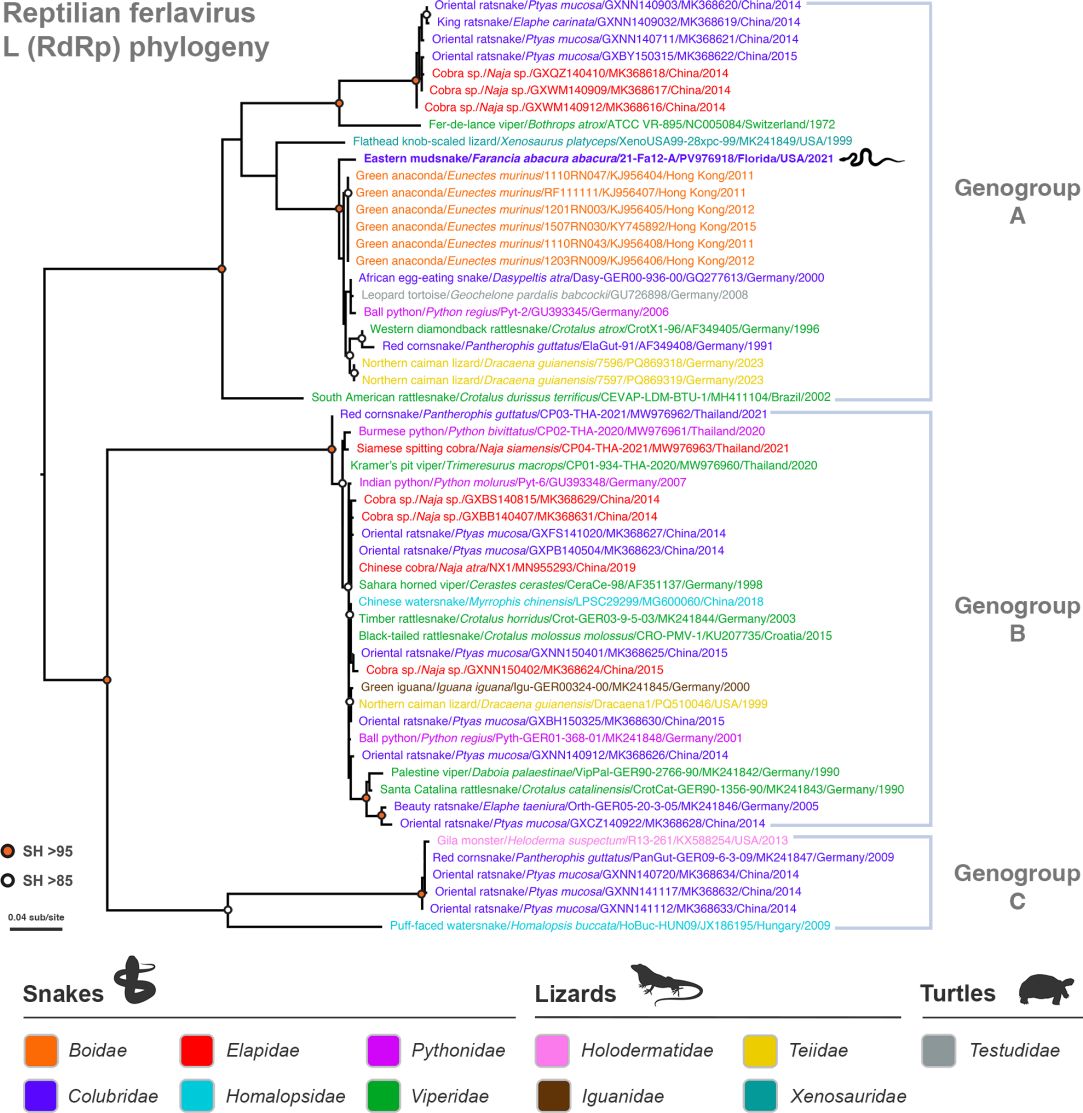
Evolutionary relationships among the eastern mudsnake ferlavirus and reptilian ferlaviruses from zoological or other husbandry settings. A maximum likelihood phylogeny based on partial L (RNA-dependent RNA polymerase; RdRp) nucleotide sequences was inferred using ferlaviruses isolated or detected in reptilian hosts from Asia, Europe, South America, and North America. Sequences are color-coded according to family. The three major genogroups of ferlaviruses are shown on the right, with the mudsnake ferlavirus (highlighted in blue bold with a snake icon) clustering with genotype A viruses. The tree is mid-point rooted for clarity only. The horizontal branch lengths are scaled according to the number of nucleotide substitutions per site. SH-like branch supports are colored according to the key. Viruses are labeled as: host common name/host scientific name/virus ID/GenBank accession number/state (where applicable)/country/detection date. Image created, in part, in BioRender (Allison, A. [2026] https://BioRender.com/1qr6g02).

The reptilian ferlaviruses are currently classified into four genogroups based on phylogenetic analysis of a portion of the L gene (10). Three of these genogroups—A, B, and C—are composed primarily of snake and lizard isolates, while a single divergent virus detected in a Hermann’s tortoise (*Testudo hermanni*) comprises the fourth reported genogroup (12, 17). The vast majority of ferlavirus sequences in the NCBI database are short L gene RT-PCR products (~450–570 nt), with only 15 ferlavirus genomes currently available for analysis. While these L gene sequences are limited in length, they presently provide the most comprehensive assessment of phylogenetic relationships. The L gene nucleotide phylogeny revealed that the eastern mudsnake clustered with the anaconda isolates from Hong Kong, along with viruses from snake, lizard, and tortoise species imported into Germany from 1991 to 2023, with strong support (Fig. 2). This clade fell into a larger group containing the prototype FDLV from Switzerland, lizard and rattlesnake viruses from the Americas, and elapid (cobra) and colubrid (ratsnake) viruses from China, which together constitute genogroup A (2, 28–30). Genogroup B viruses were detected in snake species from five different families from Europe and Asia, while Genogroup C, the most limited genogroup in terms of currently available sequences, included viruses from snakes sampled in Europe and Asia, along with a Gila monster (*Heloderma suspectum*) from the United States (18) (Fig. 2). Comparison of the short L nucleotide sequence phylogeny to the full genome phylogeny comprised of 15 viruses (not shown) revealed the same genogroup clustering of viruses.

### U protein expression and inclusion body formation in infected cells

**Fig 3:**
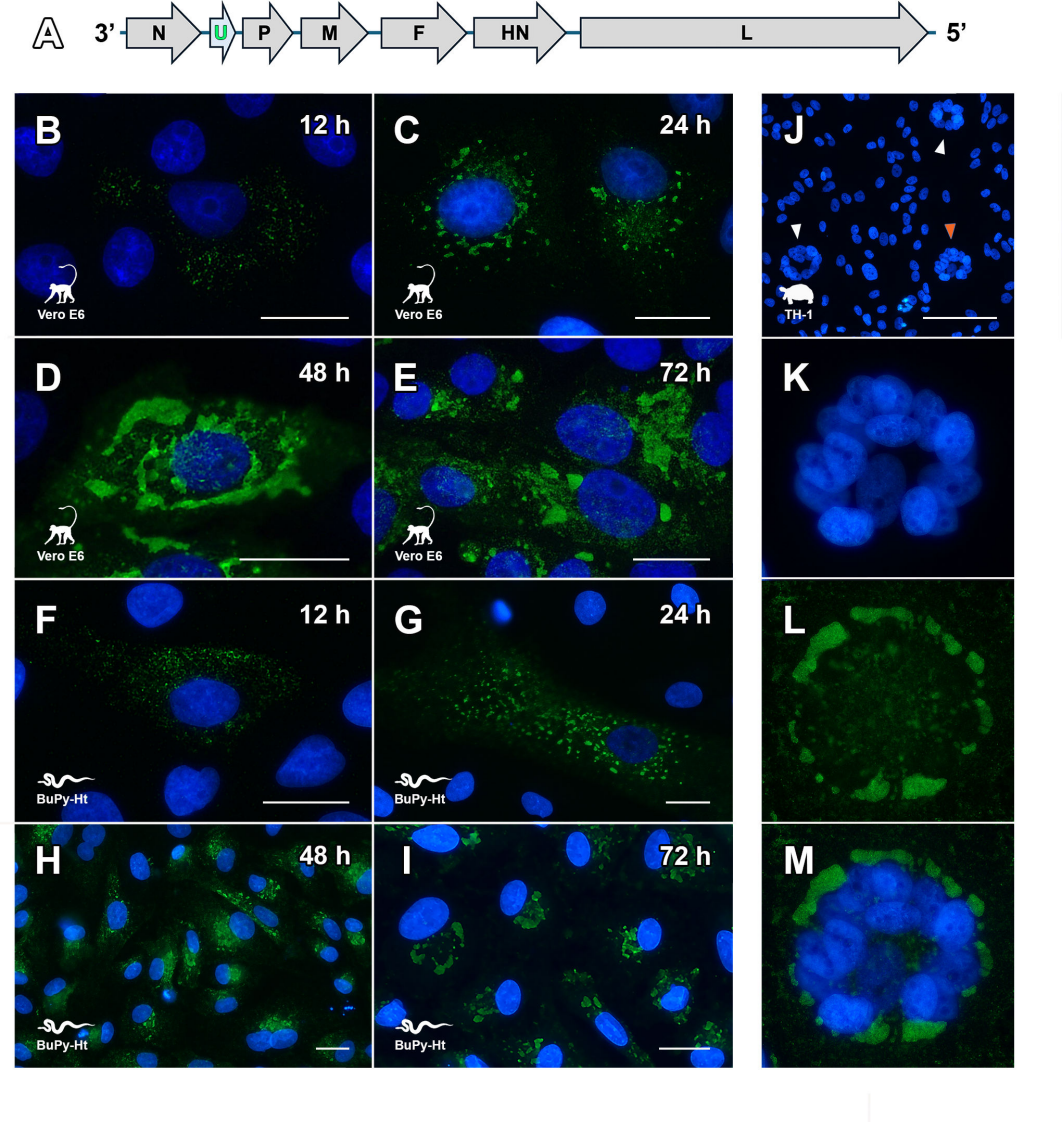
U protein expression in ferlavirus-infected mammalian and reptilian cells. (**A**) Genomic organization of the mudsnake ferlavirus highlighting the U gene located between the nucleocapsid (N) and phosphoprotein (P) genes. (**B–I**) Time course and morphogenesis of U protein expression in cells infected with the mudsnake ferlavirus. Scale bars = 25 µm. (**B–E**) Vero E6 and (**F–I**) Burmese python heart (BuPy-Ht) cells infected with mudsnake ferlavirus at 12, 24, 48, and 72 h time points (see Results for details). (**J–M**) Concentric U protein formation in association with syncytia in ferlavirus-infected Eastern box turtle (TH-1) heart cells. (**J**) TH-1 cells infected with mudsnake ferlavirus. Syncytia are indicated with arrowheads. Close-up views of the syncytium indicated with the orange arrowhead are shown in panels K–M. Scale bar = 250 µm. (**K**) A large syncytium containing 16 nuclei. (**L**) U protein localization to the periphery of the condensed nuclei, forming a ring-like structure. (**M**) Overlay between panels K and L showing the close association of the U protein with the condensed nuclei within the syncytium. All panels were stained with a rabbit anti-ferlavirus U protein primary antibody and a goat anti-rabbit IgG Alexa Fluor 488 conjugated secondary antibody and counterstained with DAPI. Image created, in part, in BioRender (Allison, A. [2026] https://BioRender.com/idge3on).

While the U protein of ferlaviruses is unique among the paramyxoviruses, juxtaposed between the N and P genes (Fig. 3A), its function or spatiotemporal expression during infection has never been determined. When using the rabbit anti-U protein polyclonal antibody developed against the mudsnake ferlavirus, the U protein was shown to localize to inclusion body-like formations over time. This was demonstrated in Vero E6 African green monkey (*Chlorocebus aethiops*) kidney cells that are deficient in interferon signaling (31), which may account for the noted robust replication (see below), as well as BuPy-Ht cells, a primary heart cell culture derived from a Burmese python (32), which are more representative of a normal host cell environment. Early in infection (12 h) in BuPy-Ht or Vero E6 cells, U protein expression could be visualized widely scattered throughout the cytoplasm of the cell as pinpoint foci or small aggregates (Fig. 3B and F). However, by 24 h, these small aggregates condensed into larger formations reminiscent of inclusion bodies of other paramyxoviruses, such as mumps virus and measles virus, which often localized in close proximity to the nucleus (33, 34) (Fig. 3C). By 48 h post-infection, more morphologically elaborate structures containing the U protein were observed, which began to occupy larger proportions of the cytoplasm of the cell (Fig. 3D and H). By 72 h post-infection, U protein expression often became further condensed at perinuclear areas, as particularly evident in the transition between 48 and 72 h in BuPy-Ht cells (Fig. 3H and I).

A common cytopathological effect of paramyxovirus infection in cell culture is the formation of syncytia or multinucleated cells (Fig. 1G), a consequence of the fusion of neighboring cell membranes by surface expression of F and HN, with fusion occurring at physiological pH (35). While syncytia formation was observed in all cell lines we infected with the mudsnake ferlavirus, it was especially prominent in Eastern box turtle (*Terrapene carolina*) heart (TH-1) cells, where syncytia tended to form ring-like structures composed of nuclei (Fig. 3J and K). Strikingly, the U protein localized within the syncytia to form circular structures that surrounded the ring of nuclei (Fig. 3L and M).

### U protein expression and inclusion body formation in transfected cells

**Fig 4:**
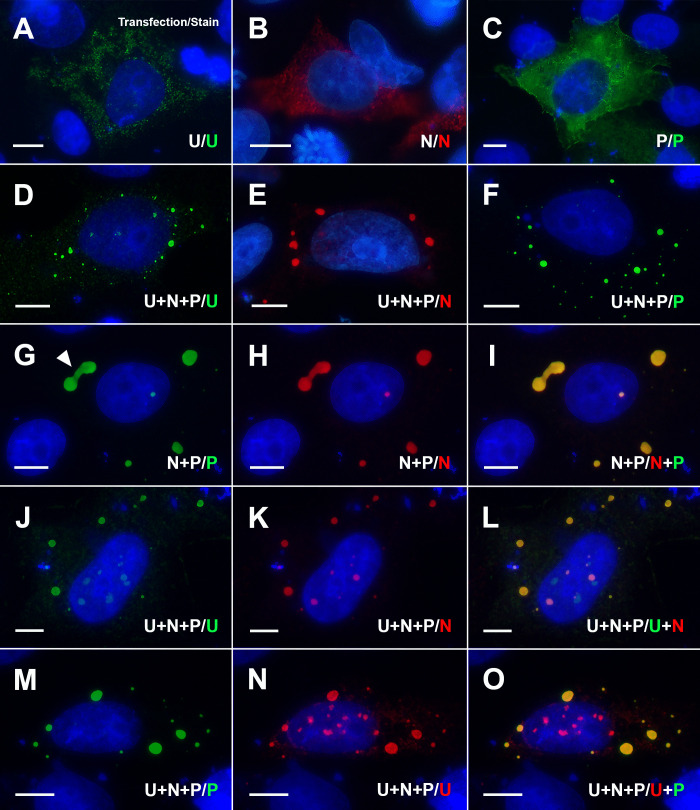
U protein association with nucleocapsid (N) and phosphoprotein (P)-mediated inclusion bodies in plasmid-transfected cells. In each panel, the plasmid(s) transfected into Vero E6 cells/antibodies used for staining and color of fluorescence are indicated. (**A–C**) Single transfections with plasmids expressing the mudsnake ferlavirus proteins U, N, or P. (**D–F**) Triple transfection with U, N, and P plasmids followed by staining for individual proteins demonstrates that all three proteins associate with spherical inclusion bodies, although a mixture of large spherical inclusions and smaller aggregates can be visualized in U protein-stained cells (**D**). (**G–I**) Co-transfection of N and P plasmids results in the formation of N-P inclusions via liquid-liquid phase separation as previously observed in other paramyxoviruses (33, 34, 37). Note the apparent separation of two inclusion bodies (arrowhead in panel **G**), indicating the fluidity of their formation. Triple transfection with U, N, and P plasmids followed by staining for U and N (**J–L**) or U and P (**M–O**) demonstrates that spherical inclusions contain both U and N (panel L overlay) and U and P (panel O overlay). (**A–O**) N expression was detected using a mouse anti-His tag monoclonal antibody conjugated with Cy3 (panels **B**, **E**, **H**, **I**, **K**, and **L**). P expression was detected using a mouse anti-Flag tag monoclonal antibody conjugated with iFluor 488 (**C**, **F**, **G**, **I**, **M**, and **O**). U protein expression was detected with a rabbit anti-ferlavirus U protein primary antibody and a goat anti-rabbit IgG Alexa Fluor 488 conjugated secondary antibody (**A**, **D**, **J**, and **L**) or a goat anti-rabbit IgG Cy3 conjugated secondary antibody (**N and O**). All panels were counterstained with DAPI, with scale bars = 10 µm.

To determine whether the U protein was sufficient to create inclusion body-like aggregates alone, or if other proteins such as N or P (which are known to create inclusions) (34, 36, 37) were needed for their formation, we transfected cells with plasmids encoding the three proteins. Vero E6 cells were chosen for this analysis as they had the highest level of transfection efficiency. After transfection, U protein expression alone in Vero E6 cells led to the formation of small pin-point aggregates (Fig. 4A). Larger cytoplasmic globules, as observed in virus-infected cells (Fig. 3C through E), were not detected when U was expressed alone. Similar results were obtained when transfecting N or P plasmids individually, with widespread fluorescence throughout the cytoplasm without apparent inclusion body formation (Fig. 4B and C). In contrast, when cells were transfected with all three plasmids (N, P, and U), circular inclusion bodies that were smooth in appearance were routinely observed (Fig. 4D through F). To first confirm the inclusions observed contained both N and P together, an overlay (Fig. 4I) between N (Fig. 4H) and P co-transfected cells (Fig. 4G) showed a direct association between the two proteins. Next, in triple transfections with U, N, and P plasmids, cells were stained against U and N (Fig. 4J through L) or U and P (Fig. 4M through O). In each case, there was a direct association of U with either N (Fig. 4L) or P (Fig. 4O) in cytoplasmic inclusions, although additional independent pin-point U staining was visible in the cytoplasm as well as globule formations in (and/or above) the nucleus. Highly cross-adsorbed goat anti-rabbit Alexa Fluor 488-labeled or cross-adsorbed goat anti-rabbit Cy3-labeled secondary antibodies used to detect the rabbit anti-U polyclonal antibody showed no detectable staining against the mouse monoclonal antibodies (Fig. S1).

### mRNA expression of V/I/P site

Ferlavirus genomes normally encode for the non-essential V protein, rather than the essential P protein (23). Production of the P protein—which acts as a cofactor for RNA synthesis by the L protein (38)—is therefore completely dependent upon mRNA editing of the V gene sequence during transcription, whereby the insertion of two additional non-templated Gs into the mRNA by the polymerase is required for P production (Fig. S2). If a single non-templated guanosine is added, then a protein designated as W, I, or D (nomenclature dependent upon the virus) is produced (Fig. S2). Among the orthoparamyxoviruses, the functions of the V or W/I/D proteins have been best studied in the henipaviruses, whereby they primarily act as virulence factors involved in evading or suppressing host immune responses (39–41). In the case of ferlaviruses, the W/I protein’s unique C-terminal domain is extremely short (two amino acids) (Fig. S2), suggesting it may not have a biological role, and its existence may merely be as an intermediate by-product in an attempt to produce a P protein. However, it is possible that production of its N-terminal region may have an undescribed function (42).

To examine mRNA expression from the P ORF editing site, in particular against other ferlaviruses previously examined (9, 23), we analyzed three different cell lines infected with the mudsnake ferlavirus: two from snakes (VH2 and BuPy-Ht), along with Vero E6 cells. By analyzing mRNA expression across three cell lines, we sought to possibly identify trends in the proportion of V, P, and I mRNAs being synthesized. Analysis of 40 T/A clones from each cell line (120 clones total) (Fig. S2) demonstrated that (i) collectively, edited transcripts with more than +2 inserted guanosines were relatively rare, accounting for only 10.8% (13/120) of analyzed mRNAs, and hence, mRNAs with +0-2 G insertions dominated (89.2%; 107/120) (*P* <0.001); (ii) in terms of the different mRNAs produced from the +0-2 G transcripts sequenced (107), P (+2) was the most abundant transcript observed in all three cell lines, although this was not statistically significant (*P* = 0.2778); and (iii) although the I mRNAs (+1 or +4 G) were the least abundant of the three mRNAs produced, they were detected at nearly three times higher frequency (19/120; 15.8%) than in previous studies with viruses that normally produce V, which was more on par with the I/W proteins of viruses that normally produce P (8.5%–24.0%) and have an ascribed function for I/W (42).

### Relative infectivity and replicative fitness

**Fig 5:**
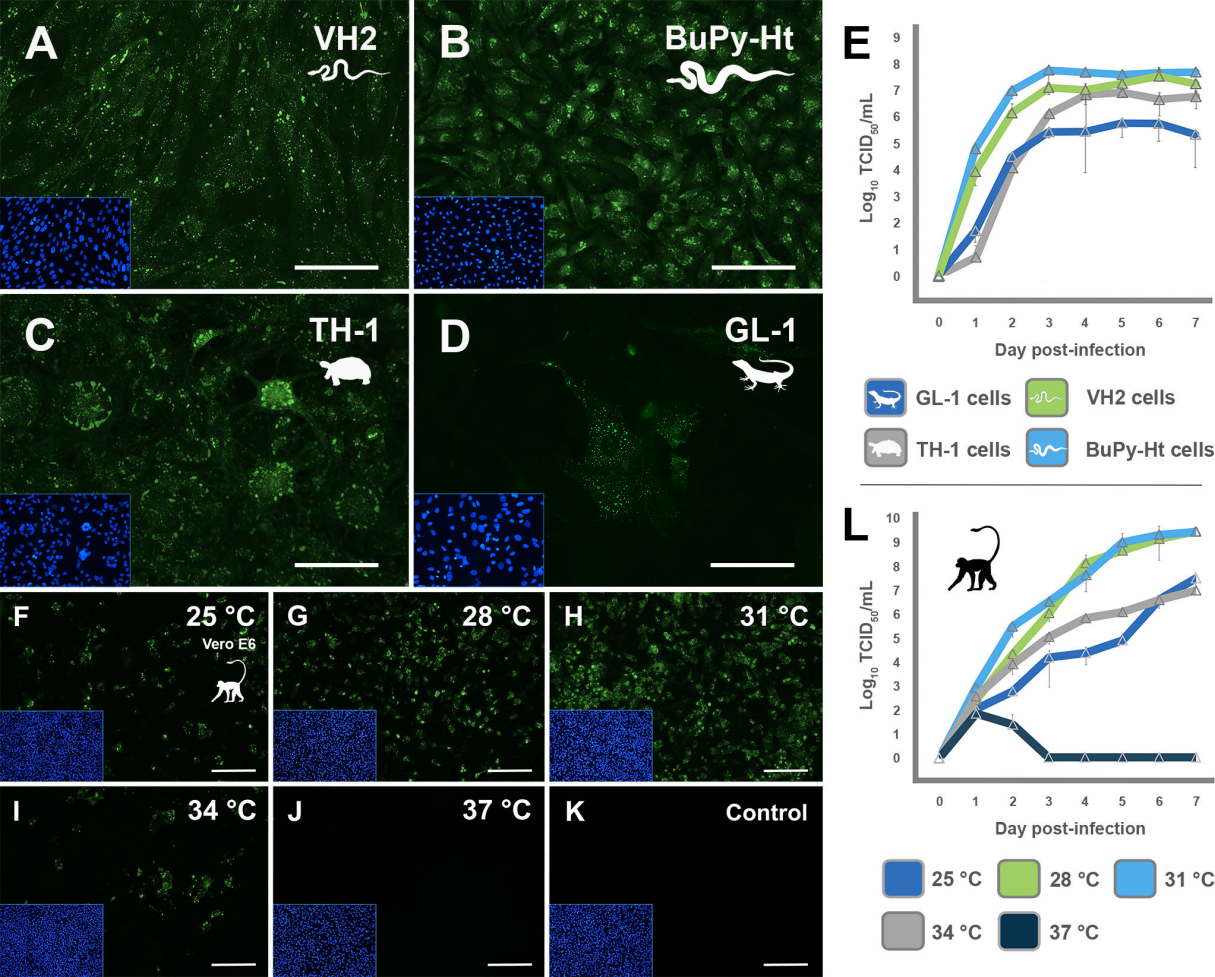
Host range and temperature-dependent fitness of the mudsnake ferlavirus in reptilian and mammalian cells. (**A–D**) Relative infectivity of the mudsnake ferlavirus in four reptilian cell lines—VH2 (Russell’s viper heart), BuPy-Ht (Burmese python heart), TH-1 (Eastern box turtle heart), and GL-1 (Tokay gecko lung). Cells were infected at a multiplicity of infection (MOI) of 1.0 at 28°C, fixed at 72 h post-infection, and stained with a rabbit anti-ferlavirus U protein primary antibody and a goat anti-rabbit IgG Alexa Fluor 488 conjugated secondary antibody and counterstained with DAPI. Corresponding images of DAPI-stained nuclei are shown as an inset to visualize the confluent monolayer. Scale bar = 250 µm. (**E**) Overlay of multi-step growth curves of cell lines in A–D that were infected at an MOI of 0.1 at 28°C and harvested daily for 7 days. Viral titers were determined by immunofluorescent TCID_50_ assay using antibodies described in sections A–D. Experiments were performed in triplicate with error bars showing standard deviations. (**F–K**) Temperature-dependent replication of the mudsnake ferlavirus in mammalian cells. Immunofluorescent assays of infected Vero E6 cells incubated at (**F**) 25°C, (**G**) 28°C, (**H**) 31°C, (**I**) 34°C, and (**J**) 37°C. Non-infected Vero E6 cells are shown in panel **K**. Monolayers were infected at an MOI of 0.1 and fixed and stained at 72 h post-infection as in panels **A–D**. Immunofluorescent images in panels F–K are from experiments independent from panel L. Scale bar = 500 µm. (**L**) Growth curve kinetics of mudsnake ferlavirus in Vero E6 cells at five temperatures (25°C, 28°C, 31°C, 34°C, and 37°C) over 7 days. Vero cells were infected at an MOI of 0.1 and harvested and titrated as in panel E. Note replication is inhibited at 37°C. Experiments were performed in triplicate with error bars showing standard deviations. Image created, in part, in BioRender (Allison, A. [2026] https://BioRender.com/jsm5oy8).

As ferlaviruses have been isolated from a variety of species across the class *Reptilia*, we analyzed the relative infectivity of the mudsnake isolate using snake, turtle, and lizard cell lines. Three of the cell lines used—BuPy-Ht, TH-1, and GL-1—represent species (i.e., Burmese python, Eastern box turtle, and Tokay gecko [*Gekko gecko*], respectively) that are present in the Florida Everglades and thus provide a representative collection of reptilian host species that the virus could potentially encounter in nature. Relative infectivity assays demonstrated that all species were susceptible to infection (Fig. 5A through D). Based on immunofluorescence, infection was widespread in both snake and turtle (heart) cells by 72 h post-infection (Fig. 5A through C), but was more restricted in Tokay gecko (lung) cells (Fig. 5D). Multi-step growth curves largely mirrored the relative infectivity studies, with reduced replication rates and lower titers observed in Tokay gecko cells (Fig. 5E). The two snake cell lines (VH2 and BuPy-Ht) had similar viral growth kinetics, with the virus reaching its highest mean daily titer in Burmese python cells at day 3 post-infection (log10^7.8^ TCID_50_/mL). The turtle cells had slower viral replication rates than the snake cells, in particular between 0 and 48 h post-infection (Fig. 5E). Overall, replicative fitness was highest in Burmese python cells, followed by Russell’s viper, Eastern box turtle, and lastly Tokay gecko cells (Fig. 5E).

### Temperature-dependent infectivity

Ferlavirus replication is known to be inhibited at normal mammalian temperatures (37°C) (43, 44). However, the comparative replicative fitness of ferlaviruses at varying temperatures has not been previously analyzed. We infected Vero E6 cells (which are tolerant of a wide range of temperatures) at 3°C intervals from 25°C to 37°C (Fig. 5F through J). The virus was shown to replicate only at temperatures below the normal body temperature of mammals (<37°C), with virus replication being optimal between 28°C and 31°C and comparatively reduced at 25°C and 34°C (Fig. 5L). Virus replication was completely inhibited at 37°C, with no virus detected beyond 48 h post-infection at 37°C (Fig. 5L), suggesting that the temperature of a snake (in the wild or in captivity) could have a major effect on the outcome of ferlavirus infection, with higher temperatures restricting replication, and presumably pathogenesis.

### A hypersalivating Burmese python and the identification of a novel hepacivirus with a putative additional cysteine protease

**Fig 6:**
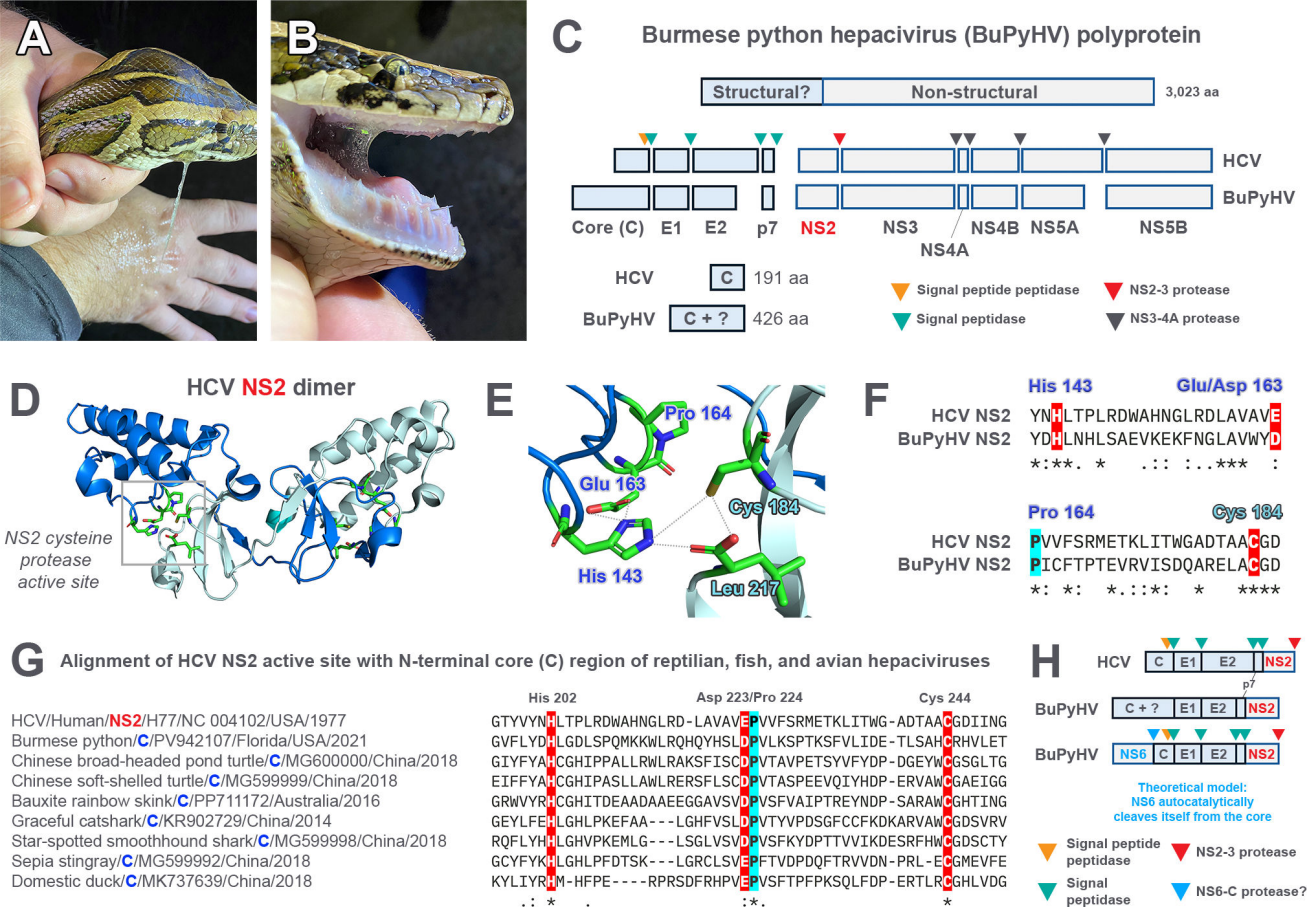
Burmese python hepacivirus contains a putative NS2-like cysteine protease in its N-terminus, which is conserved among early vertebrate hepaciviruses but absent in mammalian hepaciviruses. (**A–B**) A 2.3 m, 6.6 kg adult male Burmese python, captured near the mudsnake ferlavirus outbreak site, exuded excessive amounts of saliva (hypersalivation), a condition often observed in respiratory (viral) infections. Photos courtesy of Matthew Kogo, published with permission. (**C**) RNA-Seq from liver RNA of the python revealed the complete polyprotein of a novel hepacivirus, tentatively designated as Burmese python hepacivirus (BuPyHV). A comparison of the predicted cleavage products (individual proteins) of the BuPyHV polyprotein relative to those experimentally determined for hepatitis C virus (HCV) is shown. Proteases involved in the cleavage of the HCV polyprotein are designated by colored triangles. Noted size differences of predicted BuPyHV proteins in relation to HCV included a truncated E2 glycoprotein and NS5A, along with an extended core (**C**) region containing an additional 235 aa. (**D**) Crystal structure of the HCV NS2 dimer (pdb_00002hd0), which contains a cysteine protease active site (box) formed via each of two individual monomers, is shown in marine and pale cyan. (**E**) The HCV NS2 active site contains the catalytic triad of His^143^, Glu^163^, and Cys^184^, with Pro^164^ being conserved among all HCV isolates (47). Leu^217^ is the very C-terminal residue of NS2 that is coordinated with the active site (replaced with an Asn in BuPyHV based on alignments). (**F**) Alignment of the NS2 active site between HCV and BuPyHV. Note glutamic acid (Glu^163^) is often substituted for aspartic acid (Asp) in active site motifs for cysteine proteases. (**G**) Alignment of HCV NS2 active site with the N-terminal extended core (**C**) region in BuPyHV revealed the presence of an NS2-like catalytic triad, including the conserved Pro adjacent to the Asp/Glu residue, suggesting a cysteine protease function. Numbering is based on the BuPyHV N-terminal region. Analysis of hepacivirus sequences from NCBI containing extended N-terminus core regions demonstrated that the active site motif is well conserved among viruses from lower vertebrate hosts, including cartilaginous fish, reptiles, and a single divergent avian hepacivirus clade (see Fig. 7). (**H**) Theoretical configuration of the N-terminal region of BuPyHV. Only the first five genes of the genomes of BuPyHV and HCV are shown for clarity. Homologous to the N^Pro^ of pestiviruses (48), the N-terminal region of BuPyHV may contain a nonstructural protein (designated as NS6) with a cysteine protease motif that cleaves itself from the core and remaining polyprotein. However, the putative catalytic activity and function of NS6 remain to be experimentally verified.

Just prior to the initiation of our Burmese python swabbing project to search for ferlaviruses, a captured adult male Burmese python was shown to be actively hypersalivating (Fig. 6A and B), a clinical sign often observed with respiratory (e.g., viral) infections, notably ferlaviruses (45) and serpentoviruses (46). As such, a full post-mortem evaluation was performed on this python. The oral cavity contained abundant blood admixed with thick, viscous, clear exudate (the blood was attributed to the euthanasia method). No other gross lesions were observed. Histological lesions were observed in the liver and lungs. The liver contained marked, diffuse hepatocellular lipid-type vacuolation comprised of cytoplasmic expansion by one to several large, clear, well-demarcated vacuoles. There was also mild, acute, multifocal pulmonary edema with occasional mild foveolar thickening and frequent, widely scattered, prominent eosinophilic granulocytes within capillaries along the foveolar epithelium.

Total RNA was extracted from individual tissues (lung, liver, gall bladder, gastrointestinal tract, kidney, splenopancreas, heart, in addition to oral and vent swabs), pooled together, and deep sequenced (RNA-Seq). Protein BLAST analysis of translated contigs revealed two viruses within the pooled sample: a serpentovirus, similar to those previously detected in Burmese pythons from South Florida (see below) (32), and a novel hepacivirus (family *Flaviviridae*, genus *Hepacivirus*). As hepaciviruses are hepatotropic, we specifically targeted the liver for further RNA-Seq, resulting in the assembly of a complete polyprotein sequence of 9,069 nt, a length typical of most hepaciviruses, although with a notably extended N-terminal region (426 aa) relative to the prototype hepatitis C virus (HCV), which normally encodes the core (C) protein (191 aa) (Fig. 6C). The polyprotein showed the highest amino acid identity, albeit only ~35%, to two other reptile hepaciviruses—Chinese broad-headed pond turtle hepacivirus (MG600000) and Chinese softshell turtle hepacivirus (MG599999) (49)—and also shared similar amino acid identities (~31%–32%) to rodent hepaciviruses from a bank vole (*Myodes glareolus*; KC411777) (50) and Chinese pygmy dormouse (*Typhlomys cinereus*; PQ677983) (51). Due to its significant divergence from known hepaciviruses (i.e., p-distance >0.25 in NS3 and >0.3 in NS5B) (52), it was tentatively designated as a novel virus species, Burmese python hepacivirus (BuPyHV).

Structural modeling analysis of the unique N-terminal region of the BuPyHV polyprotein showed that aa residues 166–238 contained strong homology (92.9% confidence) to the HCV NS2-3 protease (pdb_00002hd0) (Fig. 6D and E). Protein BLAST analysis further revealed a ~29%–35% aa identity spanning BuPyHV residues 150–274 to various flaviviruses, aligning to the conserved NS2-3 protease domain in both hepaciviruses and pegiviruses. The BuPyHV N-terminal region has the same active site motif as the NS2-3 protease of hepaciviruses (including its own), that is a prerequisite for cysteine protease activity: a histidine (H; His), cysteine (C; Cys), and a negatively charged amino acid, either a glutamic acid (E; Glu) or aspartic acid (D; Asp) (47, 53) (Fig. 6E through G and 7). In addition, a proline (Pro^164^ in HCV) that lies adjacent to the glutamic acid (Glu^163^)—which is conserved among HCV isolates and thought to bend the peptide backbone adjacent to Glu^163^ to allow for catalysis (47)—was also present (Fig. 6E through G). As most hepacivirus core (capsid) proteins are small (≤200 aa), these findings suggest that the N-terminal region of BuPyHV (426 aa) may contain a C protein followed by an N-terminal protein (or possibly proteins) that we provisionally designate as nonstructural protein 6 (NS6) (Fig. 6H). Predicted transmembrane domains were not found in NS6, and as a nonstructural N-terminal cysteine protease (N^Pro^) that precedes the structural genes is present in pestiviruses (48), in addition to the amino acid identity of NS6 to NS2 (suggesting it may have evolved by gene duplication), it was given the provisional designation as a nonstructural protein. Based on alignments with HCV NS2/NS3, the predicted cleavage site between NS6 and C is ^276-^RA/GP^−279^, which would result in NS6 and C proteins of 277 (31.5kDa) and 149 (16.1kDa) amino acids in length, respectively. Protein BLAST of NS6 with the protease domain removed (residues 1–165) did not reveal any identity to known viral or cellular proteins.

**Fig 7:**
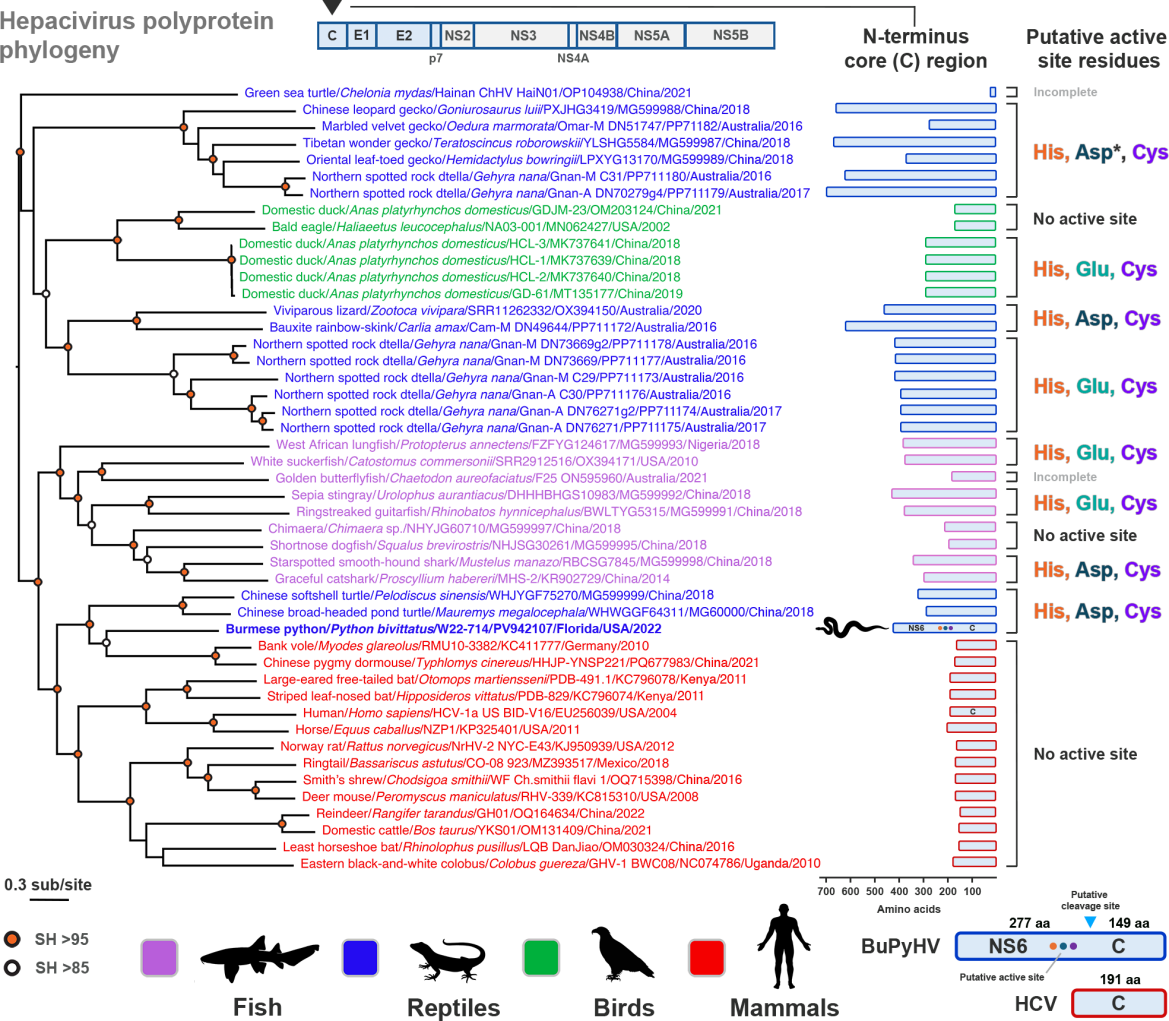
Evolutionary relationships of Burmese python hepacivirus (BuPyHV) to reptilian, fish, avian, and mammalian hepaciviruses and a comparison of their N-terminal core regions. A maximum likelihood phylogeny was inferred using polyprotein amino acid sequences of hepaciviruses from Africa, Australia, Asia, Europe, and North America. Sequences are color-coded according to vertebrate class. BuPyHV is highlighted in bold blue and with a python icon. The N-terminal core region, which is extended (relative to mammalian hepaciviruses) in fish, reptilian, and some avian viruses, is highlighted on the right and shown to approximate scale in length. The presence or absence of a NS2-like catalytic triad (i.e., histidine [His], aspartic acid/glutamic acid [Asp/Glu], and cysteine [Cys]) in the N-terminal region of all viruses is shown. An Asp asterisk (*) in the Australian and Chinese clade of geckos denotes that, while the active site is present, the conserved proline adjacent to the catalytic Asp/Glu is absent (see Fig. 6). Proline is present in all other clades containing the active site motif. Note for sequences which are denoted as containing “no active site,” a 5′ UTR was present, suggesting the full polyprotein has been sequenced. For samples deemed “incomplete,” a 5′ UTR was absent, and thus the presence or absence of the active site is uncertain based on available data. The NS6 protein and the relative location of its putative active site, along with the predicted cleavage site between NS6 and C, are shown in comparison to the HCV core (C) protein (bottom right). The tree is mid-point rooted for clarity only. The horizontal branch lengths are scaled according to the number of amino acid substitutions per site. SH-like branch supports are colored according to the key. Viruses are labeled as follows: host common name/host scientific name/virus ID/GenBank accession number/state (where applicable)/country/detection date. Image created, in part, in BioRender (Allison, A. [2026] https://BioRender.com/6ndwohp).

Phylogenetic analysis of the complete polyprotein demonstrated that BuPyHV grouped (although with a relatively long branch) with the rodent viruses from a bank vole and Chinese pygmy dormouse, which, in turn, formed a sister group to the turtle hepaciviruses (Fig. 7). To determine the extent to which the putative NS6 active site could be found in other hepaciviruses, we mapped its presence/absence onto the polyprotein phylogeny containing a comprehensive collection of 47 vertebrate hepaciviruses. The putative catalytic triad was shown to be well conserved among divergent hepaciviruses, particularly among those from lower vertebrates (i.e., reptiles and cartilaginous/bony fish) (Fig. 6G and 7). The active site motif was found in all reptilian hepaciviruses analyzed, although a single clade of geckos from Australia and China contained the active site without a proline adjacent to the catalytic aspartate (Fig. 7) (54). The catalytic triad, while found in most fish viruses, was absent in a shortnose dogfish (*Squalus brevirostris*; MG599995) and chimaera (*Chimaera* sp.; MG599997) sequence (Fig. 7). While no mammalian hepaciviruses contained an N-terminal protease active site, it was detected in a group of domestic duck (*Anas platyrhynchos domesticus*) viruses in China collected from 2018 to 2019 (yet absent in other avian hepaciviruses) (Fig. 6G and 7).

**Fig 8:**
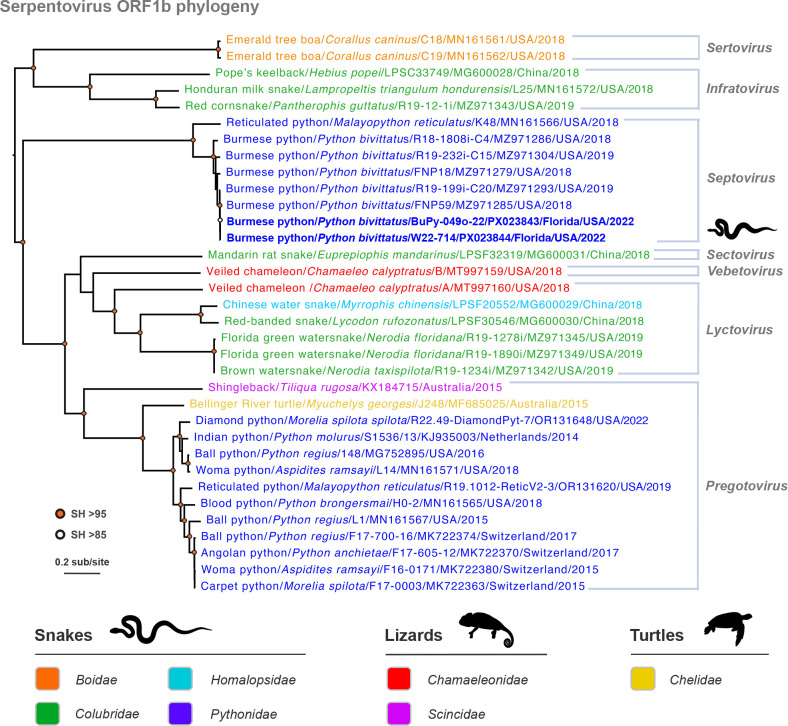
Evolutionary relationships of Burmese python serpentoviruses from South Florida to serpentoviruses from snake, lizard, and turtle species from Asia, Australia, Europe, and North America. A maximum likelihood amino acid phylogeny of ORF1b sequences of serpentoviruses (order *Nidovirales*, family *Tobaniviridae*, subfamily *Serpentovirinae*) from reptilian hosts is shown. Sequences are color-coded according to reptilian family. The subfamily *Serpentovirinae* is currently divided into seven genera, as shown on the right. Note the serpentoviruses detected from Burmese pythons in this study (shown in bold blue) cluster with strong support to other Burmese python viruses previously collected from South Florida (32), which fall into the *Septovirus* clade. The tree is mid-point rooted for clarity only. The horizontal branch lengths are scaled according to the number of amino acid substitutions per site. SH-like branch supports are colored according to the key. Viruses are labeled as follows: host common name/host scientific name/virus ID/GenBank accession number/state (where applicable)/country/detection or isolation date. Image created, in part, in BioRender (Allison, A. [2026] https://BioRender.com/fa4ummq).

The hepacivirus-infected python was co-infected with a serpentovirus (family *Tobaniviridae,* subfamily *Serpentovirinae*, genus *Septovirus*), which are positive-sense RNA viruses that are well-known pathogens of captive snakes (46). Phylogenetic analysis based on ORF1b polyprotein sequences revealed that this virus fell into a clade (with strong support) consisting of serpentoviruses previously detected from Burmese pythons in South Florida during 2018–2019 (32) (Fig. 8). Collectively, these viruses are provisionally designated as Burmese python septovirus (BuPySV). The near-complete septovirus genomes (26,812 nt) from the hypersalivating python (W22-714) and another python analyzed during our surveillance (BuPy-049; see below) (Fig. 8) showed the viruses shared 84%–97% nucleotide identity to the Burmese python septoviruses previously identified in South Florida, whereby divergent viruses found in different subpopulations of pythons may be representative of separate introductions (32).

### RNA viruses detected in Burmese pythons from the outbreak site

**Fig 9:**
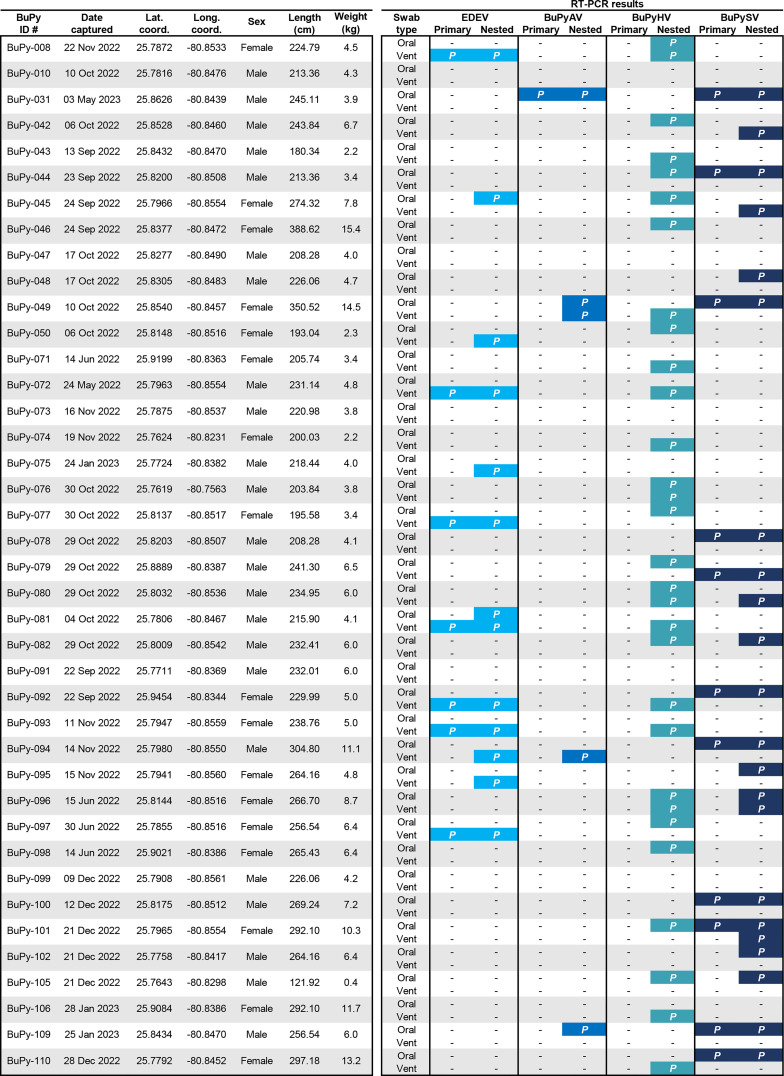
RNA viruses detected in Burmese pythons sampled in and around the ferlavirus outbreak site. RNA-Seq analysis of oral and vent swabs from 40 Burmese pythons resulted in the detection of a novel alphavirus (Eden alphavirus; EDEV) and the first North American reptilian arterivirus (Burmese python arterivirus; BuPyAV), along with additional detections of Burmese python hepacivirus (BuPyHV) and the formerly characterized serpentoviruses (Burmese python septoviruses; BuPySV). No reptilian ferlaviruses were detected in the pythons. From the RNA-Seq reads generated from pooled oral or vent samples of pythons, primers were developed for screening individual python swab RNA for the detected viruses by primary and nested RT-PCR (see Table S1). Positives are shown with a colored box and a “*P*” symbol. For each python tested, latitude and longitude coordinates are shown, along with sex, total length, weight, and date captured.

As the ferlavirus isolated from the eastern mudsnake showed high levels of nucleotide identity to viruses from captive anacondas (a boa species), this raised the possibility that it could have originated via cross-species transmission from an invasive constrictor species (i.e., boa or python) that is established in the Everglades. As Burmese pythons, an introduced species, were known to be present within the mudsnake outbreak site, we screened 40 pythons for ferlaviruses and other viral infections (Fig. 9). Although ferlaviruses were not detected in any of the Burmese pythons, we identified four viruses, three of which were new species.

**Fig 10:**
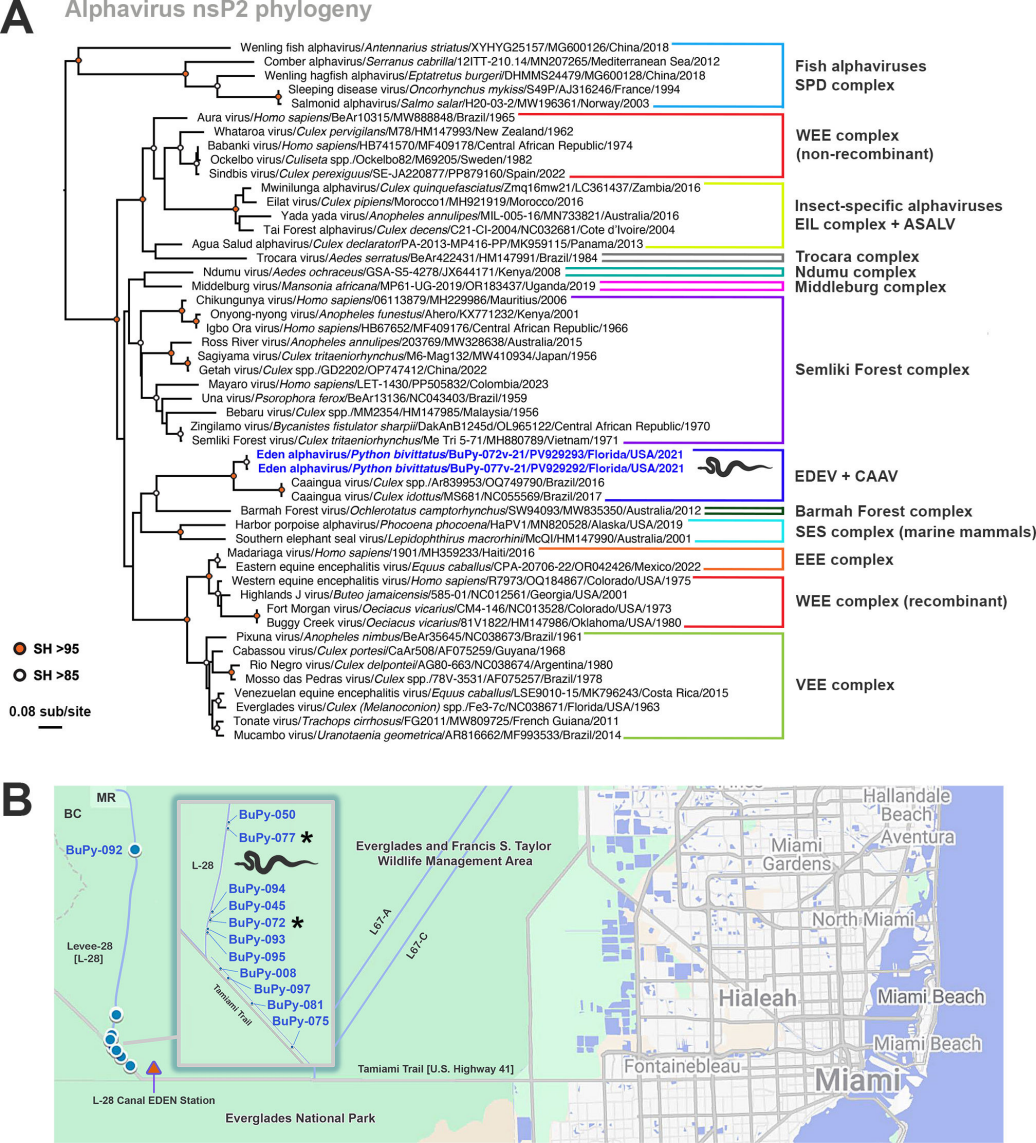
Identification of a novel alphavirus in Burmese pythons from South Florida. (**A**) Maximum likelihood phylogeny of nsP2 amino acid sequences for all known alphavirus species. Viruses are grouped according to antigenic/genetic complexes and/or as phylogenetic groups of viruses which share similar ecological or host traits (e.g., fish, marine mammal, or insect-specific alphaviruses) (59). Note the clustering of Eden alphavirus (EDEV) with Caainguá virus (CAAV) from Brazil, with strong support, which may represent a new complex. The tree is mid-point rooted for clarity only. The horizontal branch lengths are scaled according to the number of amino acid substitutions per site. SH-like branch supports are colored according to the key. Viruses are labeled as follows: virus name/host scientific name/virus ID/GenBank accession number/state (where applicable)/country/detection date; (**B**) Geographic locations of EDEV-positive Burmese pythons are shown as blue circles and are based on GPS coordinates. The majority of pythons (11/12) were found at the junction between Levee-28 (L-28) and the Tamiami Trail within the Everglades and Francis S. Taylor Wildlife Management Area, approximately 40 miles west of Miami. The inset shows an expansion of these locations, with asterisks denoting the two viruses shown in the nsP2 phylogeny in panel A. Note the location of L-28 Canal EDEN Station. BC = Big Cypress National Preserve; MR = Miccosukee Reservation; ASALV = Agua Salud alphavirus; EEE = Eastern equine encephalitis; EIL = Eilat; SDP = Salmon pancreas disease; SES = Southern elephant seal; VEE = Venezuelan equine encephalitis; WEE = Western equine encephalitis. Image created, in part, in BioRender (Allison, A. [2026] https://BioRender.com/k9i54dt). Map data © 2025 Google.

Foremost, a novel alphavirus (family *Togaviridae*, genus *Alphavirus*) was detected (Fig. 10). RT-PCR screening of individual RNA samples detected the alphavirus in 12/40 Burmese pythons (30%). The alphavirus was detected almost exclusively in the vent (11/12; 92%), with one single oral detection (1/12; 8%) and one single dual oral-vent detection (1/12; 8%) (*P* <0.001) (Fig. 9). When attempting to assemble the complete genome by RNA-Seq, the largest contig that could be built was 1,298 nt (limited to nsP2), suggesting low levels of viral RNA in our samples, which was corroborated by the maximum number of alphavirus reads (470) in the assembled contigs. Phylogenetic analysis based on nsP2 amino acid sequences demonstrated the python alphavirus was most closely related to Caainguá virus (CAAV) (Fig. 10A), a virus first identified in *Culex* mosquito pools from Brazil in 2019 (55). This new virus from Burmese pythons was tentatively designated as Eden alphavirus (EDEV), as the infected pythons were collected in close proximity to the L-28 Canal EDEN (Everglades Depth Estimation Network) station along the Tamiami Trail (Fig. 10B). Not surprisingly, due to the low levels of RNA and non-ideal transport media (RNA*later*), virus isolation attempts in BuPy-Ht, Vero E6, and C6/36 (Asian tiger [*Aedes albopictus*] larval) cells from alphavirus-positive samples were unsuccessful (56–58).

Additionally, we identified a novel arterivirus tentatively designated as Burmese python arterivirus (BuPyAV; family *Arteriviridae*). Analysis of the assembled contig (170 aa) containing nsp10 helicase gene sequences from BuPy-049 showed that it had the highest amino acid identity (50%) to Guangdong greater green snake arterivirus (YP_009755862) detected from a greater green snake (*Cyclophiops major*) in China (49). BuPyAV was detected in 10% (4/40) of pythons (oral [3/4; 75%] and vent [2/4; 50%] samples). As we were unable to extend this short sequence (170 aa) further through additional RNA-Seq, coupled with the high degree of divergence of BuPyAV sequences to nsp10 helicase sequences of other animal arteriviruses, phylogenetic analysis was not performed. After identification of the novel hepacivirus (BuPyHV) in the hypersalivating python, we screened an additional 40 pythons for this virus. BuPyHV was detected in 65% (26/40) of snakes. No preponderance of vent or oral detection was noted, as 10 were positive by vent swab only and 12 were positive by oral swab only, with four snakes positive on both swabs (Fig. 9).

Lastly, we identified Burmese python septovirus (BuPySV), a serpentovirus. Conversely to the alphaviruses, there was a preponderance for detection of serpentovirus RNA from the oral cavity over the vent, with 91% (10/11) of the primary RT-PCR positives from the oral swabs (Fig. 9). However, serpentovirus RNA was detected from vent swabs in 30% (6/20) of positive snakes, suggesting that the fecal-oral route of transmission may occur in concert with the prototypical respiratory route as previously noted by others (46, 60). Notably, multiple (two to three) viruses were detected in 57.5% (23/40) of pythons (Fig. 9). Weight, length, or sex was not statistically associated with the number of viruses detected. The most commonly observed co-detection was serpentovirus and hepacivirus (9/40; 22.5%), followed by alphavirus and hepacivirus (7/40; 17.5%). Geographical locations of all alphavirus-positive snakes based on GPS coordinates are shown in Fig. 10B.

## DISCUSSION

While ferlavirus disease outbreaks have been described in numerous snake species from zoological or private collections throughout the world (3, 4), they have not been previously reported in free-ranging snakes. As such, our observation of morbidity and mortality of numerous eastern mudsnakes over the course of several months in the Everglades represents the first documented ferlavirus outbreak recorded in wild snakes. The noted clinical signs—contorting on riverbanks with frothing oral exudate—corroborate previous observations of ferlavirus infections in managed settings, where snakes can expel blood and caseous-purulent material from the glottis, filling nasal passageways and the oral cavity (4). The major pathological manifestations we observed in wild snakes—pulmonary edema and necrotizing enteritis—are similar to infections in managed snakes where the respiratory tract is the primary target of the virus, with severe intestinal involvement less commonly observed (9, 14). Additionally, skin lesions were observed in the mudsnakes, as in fatal infections in green anacondas (9).

The source of this virus, whether it be from an invasive snake species that has been introduced into the Everglades, a native snake species, or if it has a non-snake reptilian origin, remains to be determined. In concert with the outbreak of a genogroup A ferlavirus in mudsnakes reported here, a genogroup C ferlavirus was found in a lung gavage of a single eastern indigo snake (*Drymarchon couperi*) around the same time frame in Hendry County, Florida, ~100 km north-northwest of the outbreak site (21). This suggests that multiple ferlavirus genogroups are already present and potentially established in Florida and possibly other states. Ferlaviruses may be endemic to Central and South America (19). Viral RNA has been detected in the venom gland of a wild-caught pit viper species—the jararaca (*Bothrops jararaca*)—from Brazil (20), which is the same genus of vipers linked to the original FDLV outbreak in Switzerland (2). Additionally, a number of outbreaks have been linked to reptile shipments from countries such as Peru, with viruses isolated from caiman lizards imported into France and Germany related to the virus from the mudsnake outbreak (15, 19). The isolation and characterization of additional ferlaviruses from wild-caught snakes and reptiles, particularly those that have not been placed in managed care, will help clarify how widespread the viruses are in nature and their natural host range.

Ferlaviruses are unique among the paramyxoviruses in that they have a genomic organization with a U gene located between N and P. Although U protein transcripts of FDLV are expressed at high levels in Northern blots of infected cell cultures (23), the expressed protein itself has not been visualized in cells previously. We demonstrated that U protein expression was first detected as pinpoint foci or small aggregates and then started to condense as larger pleomorphic globules that often juxtaposed the nucleus, suggesting that the U protein was being recruited to inclusion bodies, specific areas within the cytoplasm of infected cells where viral proteins and RNA congregate for replication and assembly (37). This trend was similar to that observed with other orthoparamyxoviruses such as measles virus, whereby inclusion bodies are visualized initially as small spheres and subsequently increase in size and adopt various pleomorphic shapes (34). Measles virus inclusion bodies have been shown to form via liquid-liquid phase separation, whereby proteins aggregate and condense into membraneless droplet-like organelles that separate from the surrounding medium, a process reminiscent of oil droplets in water (34, 37). Phase separation, and hence the formation of inclusion bodies, is thought to be driven largely by N and P interactions (34). Similarly, co-transfection of U with N and P resulted in the localization of U to inclusion bodies, while no such inclusions were visualized when U was transfected alone. While the exact functional role of the U protein remains to be determined, these preliminary studies demonstrate that the U protein associates with viral inclusions over time and that U interacts with N, P, and/or N-P complexes.

The close phylogenetic relationship of the mudsnake ferlavirus to isolates from green anacondas (9, 26), coupled with its seemingly high pathogenicity, could suggest infection resulted from cross-species transmission via introduced nonnative snakes. While green anacondas, a species native to South America, have been observed in the Everglades, there are currently no known breeding populations, although due to their highly aquatic and cryptic nature, an introduced population could likely go undetected for years (61). In marked contrast, the Burmese python, a species native to southern and southeast Asia, is unambiguously the most widespread and established nonnative constrictor in South Florida, and its introduction into the Everglades ecosystem has had an unprecedented, devastating effect on native wildlife (62–64). Although accurate population numbers of Burmese pythons in Southern Florida are unknown, estimates in 2010 were between tens of thousands and perhaps hundreds of thousands of pythons (61).

While we did not detect reptilian ferlaviruses in the Burmese pythons we analyzed, we did discover several new viruses, in addition to other previously identified serpentoviruses (32, 46, 60). Novel viruses included Burmese python hepacivirus (BuPyHV), which represents the first snake hepacivirus identified. The elongated N-terminal region of the BuPyHV polyprotein suggested it contained multiple proteins: the core (C) protein, which is responsible for forming the icosahedral capsid (65), along with a novel protein we tentatively designated as NS6, which was shown to contain the same catalytic triad (His-Asp-Cys) found in the NS2-3 protease (47). The amino acid identity between BuPyHV NS6 and NS2 suggests that NS6 may have evolved from gene duplication of NS2, followed by its evolution into a novel protein with currently unknown function. As the NS2-3 protease in HCV is an autoprotease that is responsible for cleaving the polyprotein at a single site—the NS2/NS3 junction (66)—the NS6 protein in BuPyHV may also be involved in a similar single cleavage event of the polyprotein (i.e., release of itself from the core). While an N-terminal protease has not been previously identified in hepaciviruses, pestiviruses encode an N-terminal nonstructural protein (N^Pro^) with cysteine protease activity. N^Pro^ autocatalytically cleaves itself from the core protein (the only cleavage event it performs) and is involved in abrogating innate immune responses within the host, such as degrading IRF-2, a transcription factor involved in regulating type I interferon synthesis (48, 67). As pestivirus infectious clones in which N^Pro^ has been deleted still replicate to similar levels (albeit they have reduced virulence) (68, 69), and pestiviruses lacking N^Pro^ have been found in nature (70), this demonstrates that N^Pro^ is a non-essential ancillary protein that acts as a virulence factor. Similarly, as we have shown that the putative N-terminal active site in BuPyHV is well conserved in ancient vertebrate classes (fish and reptiles), but is absent in mammalian hepaciviruses like HCV (which do not contain any additional genes other than those found in BuPyHV), N-terminal protease activity is unlikely to be essential for survival. It is of interest that other reptilian hepaciviruses contain even longer N-terminal regions than BuPyHV, suggesting proteins in addition to NS6 remain to be characterized, as does the putative catalytic activity and function of NS6.

This hepacivirus-infected python was co-infected with a serpentovirus, which fell into a clade with other serpentoviruses previously detected in Burmese pythons from South Florida (32). As serpentovirus-induced lesions are often observed in the upper respiratory tract in snakes, a commonly noted clinical sign is increased oral mucous secretion (46), and thus, the hypersalivation in this case was most likely related to the serpentovirus infection. However, as ~23% of the pythons we tested were RT-PCR-positive for both viruses, it is unclear whether co-infection with BuPyHV may exacerbate certain pathological conditions. In addition to the novel hepacivirus, we detected a new arterivirus, designated as Burmese python arterivirus (BuPyAV). This represents one of only six reptilian arteriviruses currently identified and the first from North America (49, 71). Similar to BuPyHV, whether BuPyAV causes disease in Burmese pythons, or if it is transmissible to native snake species or other hosts, is currently unknown, although most arteriviruses have narrow host ranges (72).

A striking discovery from our analysis was the detection of a novel alphavirus from Burmese pythons, which was tentatively designated as Eden alphavirus (EDEV). Five terrestrial alphaviruses are endemic to the United States, two of which can cause fatal disease in both humans and animals—eastern equine encephalitis virus (EEEV) and western equine encephalitis virus (WEEV)—albeit the latter has been undergoing submergence in the United States over the last three decades (73). Everglades virus (EVEV), which is endemic to South Florida where we sampled, is also zoonotic. However, it is less of a public health concern than either EEEV or WEEV, as clinical encephalitis in humans is very rare, with most infections being asymptomatic or mild (74). Lastly, Highlands J virus (HJV) and Fort Morgan virus (FMV), although non-zoonotic, can be pathogenic to birds (75, 76). Three of these viruses—EEEV, EVEV, and HJV—circulate in Florida. As FMV was the last endemic North American terrestrial alphavirus to be identified (i.e., in Colorado in 1973) (77), our recognition of EDEV in Burmese pythons represents the first new alphavirus to be discovered in the continental United States in over 50 years.

EDEV does not appear to be a chance, aberrant transmission event, as we detected the virus in a significant proportion (30%) of the pythons tested. Alphavirus infection in snakes is not unprecedented, as EEEV has been detected in cottonmouths (*Agkistrodon piscivorus*) and copperheads (*Agkistrodon contortrix*)—two native pit viper species—in Alabama, potentially suggesting that snakes could be maintaining EEEV in nature during the winter months when mosquito activity is reduced (78), although in South Florida, mosquito activity is year-round. Similarly, WEEV has been isolated previously from naturally infected snakes in the United States and Canada (79, 80). Phylogenetic analysis of a portion of the nonstructural polyprotein (nsP2) of EDEV demonstrated that it is most closely related to Caainguá virus (CAAV), first identified in *Culex* mosquito pools from Brazil in 2019 (55). The full host range of CAAV in nature is currently uncertain, as while it has the hallmarks of other insect-specific alphaviruses—in that it replicates in mosquito cell cultures, but not vertebrate cells—it appears to be able to infect primary human peripheral blood mononuclear cells, albeit not productively (55). Of the five insect-specific alphaviruses that have been previously described (i.e., Eilat virus, Tai Forest alphavirus, Agua Salud alphavirus, Mwinilunga alphavirus, and Yada Yada virus), only Agua Salud alphavirus has been detected in the Western hemisphere (Panama and Brazil) (81, 82), although it is phylogenetically distinct from CAAV (81).

Although EDEV appears to be most closely related to a potential insect-specific alphavirus (CAAV) based on available data, the high detection rate of the virus in pythons would suggest that it is replicating in the snakes and is thus a vertebrate-infecting alphavirus. There is also a possibility that the alphavirus is present in a prey item of the pythons that we are then detecting in feces. In terms of potential vectors for EDEV, certain *Culex* (*Cx.*) species (i.e., *Cx. erraticus*, *Cx. pilosis*, and *Cx. quinquefasciatus*) are known to actively feed on Burmese pythons in South Florida (83), and thus it may be prudent to investigate whether such species are infected with EDEV. It is also possible that EDEV was introduced by a tropical mosquito species (84), or via a persistent infection in Burmese pythons, similar to those observed in WEEV- and EEEV-infected snakes (85, 86), and has been circulating in the Everglades for decades. Clearly, more studies are needed to discern the host range, evolutionary relationships, ecology, and transmission dynamics of EDEV.

### Perspectives

As snakes are among the most difficult vertebrate animals to monitor, it is likely that other ferlavirus outbreaks have gone unrecognized and thus potential population impacts to eastern mudsnakes or other native snakes and reptiles in the Everglades ecosystem are currently uncertain. The evolutionary relationships between ferlaviruses circulating in wild populations and those in zoological or private collections remain enigmatic. Such relationships may be difficult to resolve, as there is potential for continuous reciprocal transmission between wild snakes and those under managed care within the reptile industry. Despite attempts at trade restrictions, 92% of all reptile species that are traded include individuals collected from the wild (87). In rare instances, nonnative traded reptiles inadvertently escape enclosures and/or are deliberately released. While the exact time frame of when Burmese pythons were first released into the Everglades is uncertain, estimates suggest there may have been a point of introduction of a small number of snakes prior to 1985 that has now led to their establishment in South Florida (88).

Predation and the subsequent loss of biodiversity in the Everglades have been the main focus of the impacts of the Burmese python on native wildlife (61, 89). However, an often-overlooked aspect of the introduction of the Burmese python and other invasive snakes into South Florida is the viruses and other pathogens they are infected with and the potential consequences of those pathogen introductions on native wildlife populations, or possibly public health. A notable example has been the recognition and geographical spread of the nonnative snake lungworm (*Raillietiella orientalis*), which has now become a major conservation concern for native snake populations and is believed to have been introduced into Florida by Burmese pythons (90, 91). While studies have begun to analyze the viral pathogens found in established python populations in the Everglades (32), or how pythons may influence disease transmission dynamics of zoonotic viral pathogens (92), the complete spectrum of viruses that invasive snakes in the Everglades are infected with, how transmissible they are to other reptile or non-reptile species, and the potential implications to the Everglades ecosystem and its wildlife remain understudied. As such, research aimed at discerning the complete virome harbored by invasive species, as well as increased surveillance in native reptiles and the timely reporting of die-offs (such as through the Herpetofaunal Disease Alert System, https://parcplace.org/species/herpdiseasealert/) should be a priority. As epitomized by the establishment of the Burmese python in the Everglades, further education on the dangers of releasing nonnative animals and the plight of native wildlife—in particular, the often-neglected native snakes—should be emphasized. One positive step toward this goal is the Florida Exotic Pet Amnesty Program (https://myfwc.com/wildlifehabitats/nonnatives/amnesty-program/), which assists individuals who desire to relinquish their exotic pets in an effort to curb the release of nonnative species. As Florida has the highest diversity of reptile species within the United States, with approximately 50 native snake species (25), it is imperative to attempt to protect this biodiversity from invasive threats.

## MATERIALS AND METHODS

### Pathological examination of snakes

Postmortem gross and microscopic examinations were conducted on the two mudsnake carcasses from the outbreak, along with a single Burmese python collected in the vicinity of the outbreak site at a later date (see below). Tissue samples including all major organ systems (brain, oral cavity, facial and salivary glands, nasal sinuses, tongue, skin, splenopancreas, lung, heart, liver, kidney, peripheral nerves, gonads, skeletal muscle, stomach, and small and large intestines [as well as trachea in the python]), and any additional tissues with gross lesions, were preserved in 10% buffered formalin for ≥48 h. Fixed tissues were routinely processed for histopathology, embedded in paraffin, sectioned at 4 µm, and stained with hematoxylin and eosin. Fresh tissue samples (lung, liver, gall bladder, gastrointestinal tract, kidney, pancreas, heart), along with oral and vent swabs, were collected from each snake for various ancillary (viral, bacterial, and fungal) testing as noted.

### Virus isolation

Fresh samples (~0.5 cm^3^) of intestine, kidney, and the spleen portion of the splenopancreas from the two mudsnakes were processed for virus isolation. Tissues were mechanically homogenized in 650 μL of Eagle’s minimum essential media (MEM) (Corning; Corning, NY) supplemented with 5% fetal bovine serum (FBS), 400 units/mL penicillin, 400 μg/mL streptomycin, and 1 μg/mL amphotericin B (4× antibiotics/antimycotics; Gibco, Thermo Fisher Scientific, Waltham, MA). Homogenized tissues were centrifuged (6,700 × *g* for 10 min), and the clarified supernatant (100 μL) was used to infect duplicate confluent monolayers in a 12-well plate format of VH2 and Vero E6 cells grown at 28°C in Leibovitz’s L-15 media (Corning) containing 10% FBS and 1× antibiotics/antimycotics without CO_2_. Cell lines were obtained from the American Type Culture Collection (ATCC; Manassas, VA). Cultures were passaged (100 μL) at weekly intervals. Wells exhibiting cytopathic effects were harvested and passaged an additional time to generate stock virus in a T-75 flask, and aliquots were frozen at −80°C prior to titration by immunofluorescence tissue culture infection dose 50 (TCID_50_)/mL (see below). For virus isolation attempts from oral and vent swabs of Burmese pythons, 20 μL of RNA*later* Stabilization Solution (Invitrogen, Waltham, MA) was inoculated into multiple wells in 12-well plates of (i) Vero E6, (ii) BuPy-Ht (kindly provided by Dr. Robert Ossiboff, University of Florida) (32), and (iii) C6/36 cells (ATCC) (93) using the procedures listed above.

### Ferlavirus RNA-Seq and genome assembly

RNA was extracted from stock virus using a QIAamp Viral RNA Mini kit with on-column DNA digestion using an RNase-free DNase kit (Qiagen; Valencia, CA). DNase-free RNA was then used to construct cDNA libraries using a NEBNext rRNA Depletion Kit v2 (New England BioLabs; Ipswich, MA). A paired-end, 2 × 150 bp sequencing run of cDNA libraries was performed using an Illumina NovaSeq 6000 platform. Illumina adapters, low-quality reads, and short reads (<30 bp) were removed from the FASTQ reads using the bbduk plugin in Geneious Prime (Biomatters; Auckland, New Zealand). *De novo* assembly was performed at a medium-low sensitivity, contigs were translated in each frame, and local BLAST was performed using a virus database containing sequences downloaded from NCBI. Once a match to reptilian ferlavirus was confirmed, the genome was obtained by both *de novo* assembly from the original contig and by using an appropriate reference sequence (anaconda paramyxovirus; GenBank accession KJ956404).

### U protein polyclonal antibody production

To examine the spatiotemporal patterns of expression of the U protein during infection with live virus, or by plasmid transfection, a rabbit polyclonal antibody against the U protein of mudsnake isolate 21-Fa12-A was generated by GenScript Biotech (Piscataway, NJ). Briefly, the U protein ORF was cloned into a pET30a vector with an N-terminal 6x His-tag, expressed in *E. coli*, and purified from inclusion bodies. Protein was purified by immobilized metal affinity chromatography, followed by Superdex 75 size exclusion chromatography prior to immunization of New Zealand rabbits. The final purified rabbit anti-U antibody was stored in sterile phosphate-buffered saline (PBS) supplemented with 50% glycerol, 0.01% Proclin 300, pH 7.4.

### Ferlavirus gene cloning and protein expression for immunofluorescence

To examine the role of the U protein in viral inclusion body formation, the mudsnake isolate 21-Fa12-A U protein gene was cloned into pcDNA3.1(+) (Thermo Fisher Scientific). The U protein gene was amplified by RT-PCR with gene-specific primers containing *NheI* and *HindIII* restriction sites, along with a flanking Kozak sequence (GCCACCATGG; U gene start codon underlined). The PCR products were purified using a QIAquick PCR purification kit (Qiagen) and subsequently T/A cloned into a pDRIVE vector using a PCR Cloning Kit (Qiagen). The ligation product was purified as above and then transformed into MegaX DH10B T1^R^ electrocompetent cells (Thermo Fisher) and plated on LB-agar with ampicillin, IPTG, and X-gal for blue/white screening. Colonies were picked and screened by PCR. Selected positive colonies were grown overnight, and plasmid DNA was extracted using the Wizard Plus SV Minipreps DNA Purification System (Promega). The pDRIVE U gene inserts were then digested with *NheI* and *HindIII* and subcloned into pcDNA3.1(+) prior to sequence confirmation.

To determine whether the U protein was localizing to inclusion bodies in conjunction with other ferlavirus proteins, or was capable of generating them on its own, the N and P gene sequences of 21-Fa12-A were cloned into pcDNA3.1(+) by GenScript and prepared as above. For the N gene, plasmids were synthesized as the native gene (no tag) and with a C- or N-terminal 6× His-tag. For the P gene, plasmids were synthesized as the native gene (no tag) and with a C- or N-terminal Flag-tag (DYKDDDDK). Various combinations of N and P were then tested, with C-terminally tagged N and P used in further experiments. Plasmids were transfected into Vero E6 cells at ~80% confluency in a 12-well plate format containing 18 mm No. 1.5 glass coverslips (MatTek Corp, Ashland, MA) using 3.0 µL of TransIT-LT1 reagent (Mirus Bio, Madison, WI) per µg of plasmid. At 72–96 h post-transfection, wells were fixed using 10% formalin, washed 3× with PBS, and incubated with a 1:500 dilution of (i) the rabbit anti-ferlavirus U protein polyclonal antibody (to detect U), (ii) a mouse anti-DYKDDDDK tag monoclonal antibody conjugated with iFluor 488 (GenScript) (to detect P), or (iii) a mouse anti-6× His tag monoclonal antibody conjugated with Cy3 (Rockland Immunochemicals, Pottstown, PA) (to detect N) in permeabilization buffer (1× PBS, 0.5% bovine serum albumin, 0.5% Triton X-100) for 1 h while shaking. The wash steps were repeated, and for wells stained with the unconjugated rabbit anti-ferlavirus U protein polyclonal antibody, they were incubated with either a (i) 1:2,000 dilution of a goat anti-rabbit IgG (H + L) highly cross-adsorbed Alexa Fluor 488 conjugated antibody (Life Technologies; Carlsbad, CA) or (ii) a 1:1,000 dilution of a goat anti-rabbit IgG (H + L) cross-adsorbed Cy3 conjugated antibody (Life Technologies) for 1 h while shaking, followed by a final wash series. Cells were then counterstained with DAPI (5 µg/mL; Thermo Fisher) for 3 min. Coverslips were mounted on slides using Vectashield Vibrance antifade mounting media (Vector Laboratories; Newark, CA) and sealed with CoverGrip (Biotium; Hayward, CA) overnight at 4°C. The following day, immunofluorescence was analyzed using a Nikon Ts2-FL inverted microscope with LED-DAPI, -FITC, and -TRITC filters and a DS-Fi3 digital camera (Nikon Instruments; Melville, NY). Fluorescent images/overlays were created in Adobe Photoshop (San Jose, CA).

### mRNA editing of the V/I/P site

Mudsnake isolate 21-Fa12-A total RNA was first extracted from infected VH2, BuPy-Ht, and Vero E6 cells using a RNeasy Plus Mini Kit with gDNA Eliminator spin columns (Qiagen). From the total RNA, polyadenylated RNA was then isolated using a PolyATtract mRNA Isolation System III kit (Promega; Madison, WI). Primers flanking the V/I/P mRNA editing site in the mudsnake ferlavirus (F: 5′-CAATAGAGAGACGCAGACTCCAAGAC; R: 5′-TATCTCGCCTACTTGTTCACTCAG) were designed to amplify a 322 bp fragment using SuperScript III with Platinum Taq (Thermo Fisher; Waltham, MA). The PCR products were purified using a QIAquick PCR purification kit and subsequently T/A cloned and purified as above. A total of 40 clones were Sanger sequenced for each cell line (120 clones total), and the mRNA editing site was examined to determine the proportion of V, I, and P mRNAs expressed in each cell line.

### Temperature-dependent replicative fitness in mammalian cells and relative infectivity and replicative fitness in reptilian cells

Vero E6 cells, grown to confluency in 12-well plates, were maintained in L-15 media with 10% FBS and 1× antibiotics/antimycotics at five temperatures without CO_2_: 25°C, 28°C, 31°C, 34°C, and 37°C. Wells were inoculated with an MOI of 0.1 TCID_50_/cell, and the virus was allowed to adsorb for 1 h at their respective temperatures. After adsorption, wells were washed 3× with cell culture PBS to remove any residual virus and then replenished with 2 mL of L-15 media with 10% FBS and 1× antibiotics/antimycotics. Wells were harvested daily for 7 days and frozen at −80°C until further processing. Experiments were performed in triplicate. Viral titers were determined by immunofluorescence TCID_50_/mL using 10-fold serial dilutions in 96-well plates incubated at 28°C. After 7 days of incubation, the titration plates were fixed and stained with the rabbit anti-ferlavirus U protein polyclonal antibody as above.

To determine the relative infectivity and host range of the mudsnake isolate for various reptilian hosts, the following cell lines were examined: (i) VH2, (ii) BuPy-Ht, (iii) GL-1, and (iv) TH-1. Cell lines were obtained from ATCC other than previously noted. All cells were grown in L-15 media supplemented with 10% FBS and 1× antibiotics/antimycotics without CO_2_. Upon reaching confluency in a 12-well plate format containing 18 mm No. 1.5 glass coverslips, cells were infected at an MOI of 1 TCID_50_/cell. At 72 h post-infection, cells were fixed, stained, and mounted. After the relative infectivity assays, cells were further analyzed for replicative fitness experiments as above.

### Ferlavirus screening of Burmese pythons

Forty Burmese pythons were captured within the Everglades and Francis S. Taylor Wildlife Management Area by FWC python contractors as part of a control program. After euthanasia, two samples per snake were taken using sterile, rayon-tipped applicator swabs (Puritan, Gullford, ME): (i) a swab of the oral cavity and (ii) a swab of the vent (cloacal/fecal) (32, 94). Oral and vent swabs were placed into separate cryovials containing 500 µL of RNA*later* Stabilization Solution and held in a cooler with ice packs in the field. In some cases, swabs were taken on freeze-thawed pythons that had been recently stored prior to the beginning of the study. For each python, the following information was recorded: (i) ID, (ii) sex, (iii) weight, (iv) length (tail, snout to vent, and total), (v) date/time of capture, and (vi) GPS coordinates. Swab samples were subsequently frozen at −20°C upon return from the field and were then sent on dry ice to the University of Florida and frozen at −80°C until further processing.

Total RNA was extracted from the entire swab using a RNeasy PowerLyzer Tissues and Cells Kit (Qiagen) and then used to create cDNA libraries as in the RNA-Seq section above, with a paired-end, 2× 150 bp sequencing run performed using an Illumina NovaSeq 6000 platform. After *de novo* assembly and BLAST analysis of the contigs from the pooled samples to identify viruses, primers were developed to screen oral and vent RNA extractions of individual pythons. For the hypersalivating python case, lung, liver, gallbladder, gastrointestinal tract, kidney, splenopancreas, and heart, in addition to oral and vent swabs, were taken for downstream RNA extraction and RNA-Seq. Primers used to screen individual oral and vent swabs for viruses detected within the pooled RNA-Seq samples are shown in Table S1. RT-PCRs were performed using Phusion DNA polymerase (Thermo Fisher Scientific) and ImProm-II reverse transcriptase (Promega) according to the manufacturer’s protocols. Nested RT-PCRs were set up using negative controls in triplicate with each run.

### Phylogenetic analysis

Evolutionary relationships of the viruses identified in this study were inferred using maximum likelihood phylogenetic trees incorporating sequences retrieved from NCBI/GenBank. Specifically, phylogenetic trees were based on (i) 55 reptilian ferlavirus L gene nucleotide sequences, (ii) 47 hepacivirus polyprotein amino acid sequences, (iii) 34 serpentovirus ORF1b polyprotein amino acid sequences, and (iv) 50 alphavirus nsP2 protein amino acid sequences. Accession numbers for all sequences used in analyses are provided in the tree figures. Sequence alignments utilized MAFFT (v7.522) employing the FFT-NS-i algorithm (95). Ambiguously aligned regions were removed using TrimAl (v1.4) (96) with a variable conservation threshold of 0.001 and a gap threshold of 0.7. Phylogenetic trees were inferred using the maximum likelihood method implemented in IQ-TREE (v1.6.12) (97) or IQ-TREE2 (v2.3.6) (98), with the best-fit substitution model selected using ModelFinder (99) (Table S2). Branch support was calculated using 1,000 ultrafast bootstraps replicates employing UFBoot2 (100) and the Shimodaira-Hasegwa-like (SH) approximate likelihood ratio test (101) within IQ-TREE2. Final phylogenetic tree figures were created in Adobe Illustrator, Adobe Photoshop, and/or Microsoft PowerPoint (Remond, WA).

### Sequence and structural analysis

Conserved viral protein motifs were examined using various online bioinformatic tools, including Quick2D from the Max Planck Institute Bioinformatics Toolkit (102), PolyPhobius (103), Protein Homology/analogY Recognition Engine (PHYRE2) V 2.2 (104), and the SWISS-MODEL homology-modeling server (105). Molecular weights were predicted using the ProtParam tool on the ExPASy server (106), and nucleotide and protein identities were determined using the percent identity matrix in Clustal Omega (107). Cleavage sites for BuPyHV were determined using SignalP 5.0 (core/E1 and E1/E2) (108) or based on the Clustal alignment with the HCV genotype 1a-H77 (NC_004102). The crystal structure of the HCV NS2 dimer was adapted from Protein Data Bank structure 2HD0 (pdb_00002hd0) using PyMOL (Schrodinger; New York, NY).

### Statistical analysis

The Shapiro-Wilk test was used to test the normality of length and weight data of snakes, and the Mann-Whitney test was used to compare the median difference of length and weight between multiple infections and single infections. The chi-squared test was used to compare the proportion of nested RT-PCR positives between oral and vent samples, as well as between males and females. The chi-square test for trend was used to analyze the proportion of V, P, and I mRNAs being synthesized with few mutations (0–2) versus more mutations (3–8). *P*-value <0.05 was considered significant. Statistical analyses were performed using MedCalc statistical software (MedCalc Software Ltd, Ostend, Belgium).

## Data Availability

The following sequences have been deposited in NCBI/GenBank: (i) reptilian ferlavirus mudsnake isolate 21-Fa12-A genome, PV976918; (ii) Eden alphavirus (EDEV) nsP2 sequences, PV929292-PV929293; (iii) Burmese python hepacivirus (BuPyHV) complete polyprotein coding cds., PV942107; (iv) Burmese python septovirus near genomic sequences, PX023843-PX023844; and (v) Burmese python arterivirus (BuPyAV) nsp10 sequence, PV982389. All relevant data of this study are provided within the article and supplemental material. All other data are available upon request from the corresponding author.

## SUPPLEMENTAL MATERIAL

Supplemental material jvi.01833-25-s0001.pdf Tables S1 and S2; Fig. S1 and S2.

ASM does not own the copyrights to Supplemental Material that may be linked to, or accessed through, an article. The authors have granted ASM a non-exclusive, world-wide license to publish the Supplemental Material files. Please contact the corresponding author directly for reuse.
