# Supplementary material for: The origins and potential cross-species transmission of paramyxoviruses and other RNA viruses between native snakes and invasive Burmese pythons in the Florida Everglades
Source: J Virol. 2026 Feb 24;100(3):e01833-25. doi: 10.1128/jvi.01833-25 (PMC13011470; doi:10.1128/jvi.01833-25)
Supplement: Supplemental material — Tables S1 and S2; Fig. S1 and S2. [file jvi.01833-25-s0001.pdf]

Table S1. Primers used to screen Burmese pythons for viruses detected in this study.

Virus	Primer sequence	Forward/Reverse	Target gene and length of RT-PCR product
Reptilian ferlavirus	CGAACTTGACAAAGCCATTAGA	Primary forward	Target gene: L protein
	TGAAGCAACCGCTGAAACC	Primary reverse	RT-PCR product size: 451 bp
	GACACATTCTTAGCATCAGCCA	Nested forward	Target gene: L protein
	ATCAGTCTCCCTTCTACTACATCAC	Nested reverse	PCR product size: 355 bp
Burmese python hepacivirus	ATGAGTGGACTAATAGGTTGCT	Primary forward	Target gene: NS4B/NS5A
	GCCAAGAGCCATCACAATC	Primary reverse	RT-PCR product size: 192 bp
	CCACTAAATCTAACATCCTCCCTG	Nested forward	Target gene: NS4B/NS5A
	AGCCATCACAATCAGAAACAGC	Nested reverse	PCR product size: 156 bp
Eden alphavirus	AACACCTGAAACCAAGTAAATC	Primary forward	Target gene: nsP1
	TACCCTTCACAGGACACGAC	Primary reverse	RT-PCR product size: 163 bp
	GGTCATATTCTCTGTGGGAGC	Nested forward	Target gene: nsP1
	TATAGGAATTTTTTCCTTTCAGGT	Nested reverse	PCR product size: 104 bp
Burmese python septovirus	CAAAGCAAAGGTCCGTAAC	Primary forward	Target gene: ORF1a
	TGGTAGTAAAGGCTGTGTGAC	Primary reverse	RT-PCR product size: 497 bp
	GCAAAGCCCAACCTAACG	Nested forward	Target gene: ORF1a
	TGGAGCCGAAGTGCGATG	Nested reverse	PCR product size: 190 bp
Burmese python arterivirus	GAACCTAAGGGTGTAGTCATAA	Primary forward	Target gene: nsp10
	CTGCTTCCAGGTGTTTGATA	Primary reverse	RT-PCR product size: 562 bp
	TGGTCGGCATAACTAGAGCG	Nested forward	Target gene: nsp10
	TTGGACGATGCTAACTGGG	Nested reverse	PCR product size: 224 bp

Table S2. Parameters used in phylogenetic analyses in this study.

Virus	Gene	Sequence type	Trimal consensus threshold	Trimal gap threshold	IQ-Tree version	Substitution model	File name
Reptilian ferlavirus	L	Nucleotide	gappyout	0.7	1.6.12	TN+F+I	Reptilian_ferlavirus_Partial_L_nt_gappyout_0.7_TN+F+I_Final_28072025
Eden alphavirus	nsP2	Amino Acid	gappyout	0.7	1.6.12	LG+I+G4	Eden_alphavirus_nsp2_AA_gappyout_0.7_LG+I+G4_Final_28072025
BuPy hepacivirus	Polyprotein	Amino Acid	gappyout	0.7	1.6.12	LG+F+R6	BuPy_hepacivirus_PP_AA_gappyout_0.7_LG+F+R6_Final_28072025
BuPy septovirus	ORF1b	Amino Acid	gappyout	0.7	2.3.6	LG+F+I+G4	BuPy_septovirus_ORF1b_AA_gappyout_0.7_LG+F+I+G4_Final _28072025

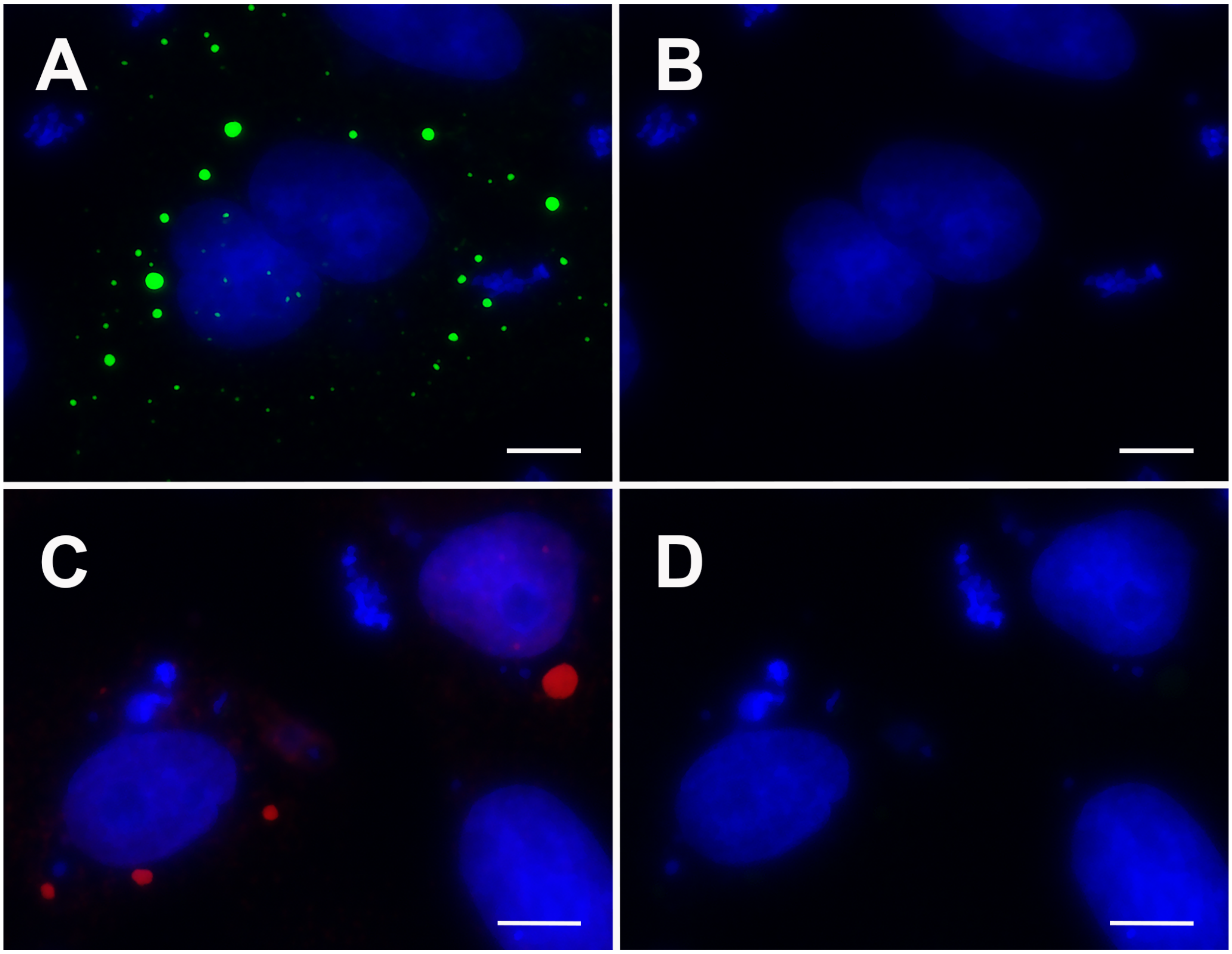

Fig S1. Control staining of goat anti-rabbit secondary antibodies (used to detect rabbit anti-U protein antibodies) against primary mouse monoclonal antibodies in transfected Vero E6 cells. (A-D) Triple transfections with plasmids expressing ferlavirus proteins U, N, and P. Cells were fixed and stained at 96 h post-transfection. Scale bars = 10µm. **(A)** Immunofluorescence (green) detecting mudsnake ferlavirus P using a mouse anti-Flag tag monoclonal antibody conjugated with iFluor 488; **(B)** Same image as A stained with a cross-adsorbed goat anti-rabbit IgG secondary antibody conjugated with Cy3 against the mouse anti-Flag tag monoclonal antibody; **(C)** Immunofluorescence (red) against mudsnake ferlavirus N using a mouse anti-His tag monoclonal antibody conjugated with Cy3; **(D)** Same image as C stained with a highly cross-adsorbed goat anti-rabbit IgG secondary antibody conjugated with Alexa 488 against the mouse anti-His tag monoclonal antibody.

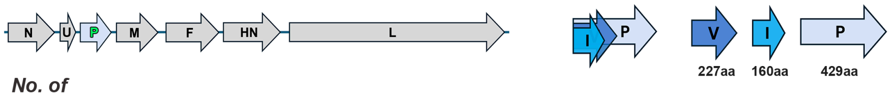

No. of NTGs	Cloned P ORF mRNA transcript	Protein produced (V, I, or P)	VH2 No. of clones	VH2 % of mRNAs	BuPy-Ht No. of clones	BuPy-Ht % of mRNAs	Vero E6 No. of clones	Vero E6 % of mRNAs	Total No. of clones	Total % of mRNAs
+0	CGGAGUAAGGGGGGCUUCUAG...	158 + 69 = 227aa V	11	27.5	11	27.5	15	37.5	37	30.8
+1	CGGAGUAAGGGGGGGCUUC*UAG*	158 + 2 = 160aa I	3	7.5	9	22.5	5	12.5	17	14.2
+2	CGGAGUAAGGGGGGGGCUUCUAG...	158 + 271 = 429aa P	21	52.5	16	40.0	16	40.0	53	44.2
+3	CGGAGUAAGGGGGGGGGCUUCUAG...	228aa V	4	10.0	2	5.0	2	5.0	8	6.6
+4	CGGAGUAAGGGGGGGGGGCUUC*UAG*	161aa I	–	–	1	2.5	1	2.5	2	1.7
+6	CGGAGUAAGGGGGGGGGGGGCUUCUAG...	229aa V	1	2.5	1	2.5	–	–	2	1.7
+8	CGGAGUAAGGGGGGGGGGGGGGCUUCUAG...	431aa P	–	–	–	–	1	2.5	1	0.8

Fig S2. **P ORF mRNA expression in reptilian and mammalian cells.** Reptilian ferlaviruses normally encode a V protein (227 aa) from the P ORF if the mRNA is unedited. However, the addition of non-templated guanosines (NTGs) at the mRNA editing site (shown in red and underlined) by the polymerase complex alters the reading frame beyond the conserved N-terminal domain (blue box), such that I (+1G; 160 aa) or P (+2Gs; 429 aa) proteins may be produced [22]. A total of 120 mRNAs from two snake cell lines – VH2 and BuPy-Ht – and one mammalian cell line – Vero E6 – were sequenced (40 clones per cell line). Note mRNAs with +0-2 G insertions were the predominant mRNAs detected (89.2%; 107/120) (*P* <0.001). In previous studies with ferlaviruses, V mRNAs dominated (68-84%) over P (11-27%), and I mRNAs were found at low levels (5.4%) [9,23,42]. In contrast, the results observed here demonstrated that of the +0-2 transcripts detected (i.e., V, I, or P mRNAs), +2 P mRNAs was the most abundant transcript detected in all three cell lines (although this lacked statistical support), suggesting there may be a degree of variability among studies on editing site mRNA transcripts, owing possibly to viral strain, cell culture system, and RNA isolation procedures, in addition to stochastic factors. Image created, in part, in BioRender (Allison, A. [2026] https://BioRender.com/c10gi91).
